# Supplementary material for: Electric pulse-tuned piezotronic effect for interface engineering
Source: Nat Commun. 2024 May 18;15:4245. doi: 10.1038/s41467-024-48451-6 (PMC11102472; doi:10.1038/s41467-024-48451-6)
Supplement: Supplementary file 1 — Supplementary Information [file 41467_2024_48451_MOESM1_ESM.pdf]

Supplementary Information for

Electric pulse-tuned piezotronic effect for interface engineering

Qiuhong Yu[1,2†], Rui Ge[1,3†], Juan Wen[1], Qi Xu[1], Zhouguang Lu[4], Shuhai Liu[1], Yong Qin[1, 5, 6]**

[1]Institute of Nanoscience and Nanotechnology, School of Materials and Energy, Lanzhou University, Lanzhou, Gansu 730000, China.
[2]Henan Key Laboratory of Photoelectric Energy Storage Materials and Applications, School of Physics and Engineering, Henan University of Science and Technology, Luoyang, Henan 471000, China.
[3]School of Advanced Materials and Nanotechnology, Xidian University, Xi'an, Shaanxi 710071, China.
[4]Department of Materials Science and Engineering, Southern University of Science and Technology, Shenzhen, Guangdong 518055 China.
[5]MIIT Key Laboratory of Complex-field Intelligent Exploration, School of Optics and Photonics, Beijing Institute of Technology, Beijing 100081, China.
[6]Advanced research institute of multidisciplinary sciences, Beijing Institute of Technology, Beijing 100081, China.
†These authors contributed equally to this work.
**Corresponding author E-mail: liushuhai1991@live.cn, qinyong@lzu.edu.cn*

This PDF file includes:

Supplementary Notes 1-21:

Supplementary Note 1 | Advantages of piezotronic tunneling junction
Supplementary Note 2 | Material selection
Supplementary Note 3 | Material characterization
Supplementary Note 4 | Piezoelectricity in wurtzite ZnO
Supplementary Note 5 | Piezoelectric nanogenerator measurement to determine the polarity of ZnO nano/microwires
Supplementary Note 6 | Effect of piezoelectric polarization on the barrier width of tunneling junction
Supplementary Note 7 | Novelty of piezotronic tunneling junction with tunable interface barrier in this work
Supplementary Note 8 | Experimental setup and electrical measurement
Supplementary Note 9 | Potential methods to achieve a long retention of the tunable barrier height
Supplementary Note 10 | Selection and impact of pulsed electric fields (intensity, frequency, etc.)
Supplementary Note 11 | In-situ measurement of tunable interfacial barrier height by AFM
Supplementary Note 12 | Reason for using low sweeping voltage
Supplementary Note 13 | Characterization of surface potential by SKPM
Supplementary Note 14 | Calculation of the strain of the devices
Supplementary Note 15 | Definition of the current change ratio

Supplementary Note 16 | Calculation of the change of effective Schottky barrier height
Supplementary Note 17 | Influence of the Ag/*n*-ZnO contact area of the side surface on the performance of the device
Supplementary Note 18 | The effect of post-annealing of as-prepared ZnO nanowires on the electric pulse-tuned piezotronic effect
Supplementary Note 19 | Theory of piezotronic effect on the tunneling junction
Supplementary Note 20 | Regulation mechanisms of interface traps/defects on electrical transport under strain
Supplementary Note 21 | Regulation mechanisms of piezoelectric charges on electrical transport under strain

Supplementary Figures 1-40:

Supplementary Fig. 1 | Influence of insulation materials on piezotronic modification of the carrier transport of piezotronic tunneling devices.
Supplementary Fig. 2 | Sensing performance of piezotronic tunneling devices.
Supplementary Fig. 3 | Morphology of HfO_2/*n*-ZnO microwire and thickness of deposited HfO_2 layer.
Supplementary Fig. 4 | Current on/off ratio of Ag/HfO_2/*n*-ZnO devices with 0.0 nm, 0.4 nm, 1.1 nm, 1.8 nm, 2.5 nm, 3.6 nm, and 7.3 nm thick HfO_2.
Supplementary Fig. 5 | Impact of the PDMS encapsulation layer on the performance of the piezotronic tunneling device by FEM simulation.
Supplementary Fig. 6 | Scanning electron microscopy (SEM), transmission electron microscopy (TEM) images, and X-ray diffraction spectra of ZnO microwire.
Supplementary Fig. 7 | Crosse-section TEM and SEM images of Ag/HfO_2/*n*-ZnO junctions and the corresponding EDX element mapping image.
Supplementary Fig. 8 | Influence of interface traps on *C*-*V* curves of Ag/HfO_2/*n*-ZnO tunneling junctions.
Supplementary Fig. 9 | Piezoelectricity in wurtzite ZnO.
Supplementary Fig. 10 | Piezoelectric nanogenerator (PENG) measurement to determine the *c*-axis orientation of the ZnO nano/microwire.
Supplementary Fig. 11 | Barrier profiles of the Ag/HfO_2/*n*-ZnO piezotronic tunneling junction under strain-free (a) and strain (b).
Supplementary Fig. 12 | Calculated potential distribution of Ag/HfO_2/*n*-ZnO tunneling junction under different strain ranged from 0.0 to 1.2 ‰ along *c*-axis.
Supplementary Fig. 13 | The basic typical structure of piezotronic devices.
Supplementary Fig. 14 | Schematic diagram for the experimental setup of the electrical measurement system.
Supplementary Fig. 15 | Electric pulse stimulation and current response of the device with tunneling junctions.
Supplementary Fig. 16 | Five recovery curves of the device with tunneling junctions under electric pulse stimulation.
Supplementary Fig. 17 | Current response and recovery characteristics regulated by the electric pulse duration.
Supplementary Fig. 18 | Electric pulse voltage used to stimulate the device.
Supplementary Fig. 19 | Electric pulse voltage and the corresponding current of the device.

Supplementary Fig. 20 | *I-V* characteristics after the electric pulse stimulation.
Supplementary Fig. 21 | Current response of the device under stimulations of electric pulse with different pulse frequency of 0.5 Hz, 1 Hz, 5 Hz, 10 Hz and 20 Hz.
Supplementary Fig. 22 | Influence of electric pulse voltage on current characteristics.
Supplementary Fig. 23 | In-situ measurement of tunable interfacial barrier height by AFM.
Supplementary Fig. 24 | Comparison of *I-V* characteristics at different sweep voltage of Ag/HfO_2/*n*-ZnO device.
Supplementary Fig. 25 | *I-V* characteristics under electric pulse stimulations with different duration at sweep voltages of ±1 V and ±5 V.
Supplementary Fig. 26 | Surface potential characterized by SKPM after electric pulse voltage stimulation.
Supplementary Fig. 27 | Histogram of surface potential statistical distribution of ZnO.
Supplementary Fig. 28 | Histogram of surface potential statistical distribution of the substrate.
Supplementary Fig. 29 | Measurement of scanning kelvin probe microscope.
Supplementary Fig. 30 | Schematic diagrams of device structure and working process.
Supplementary Fig. 31 | Comparison of *I-V* characteristics at different strain with 0 V and 80 V electric pulse voltage stimulation.
Supplementary Fig. 32 | *I-V* characteristics under the strain of 0.00%, -0.134% and no strain recovery state.
Supplementary Fig. 33 | Current as a function of the applied strain with the electric pulse stimulation.
Supplementary Fig. 34 | Ag/*n*-ZnO contacts at end/side surfaces and the equivalent circuits.
Supplementary Fig. 35 | Comparison of *I-V* characteristics under strain 0.00% and -0.1% with electric pulse stimulation.
Supplementary Fig. 36 | Characterization of the influence of annealing temperature on piezotronic properties.
Supplementary Fig. 37 | Carrier transport characteristics before and after electrical stimulation after annealing.
Supplementary Fig. 38 | Ideal MIS junction tuned by piezoelectric charges.
Supplementary Fig. 39 | Symmetric modulation of electrical transport by interface traps/defects under strain.
Supplementary Fig. 40 | Asymmetric modulation of electrical transport by piezotronic effect.

Supplementary Tables 1-2:
Supplementary Table 1 | Dielectric constants and band gaps of typical insulation materials.
Supplementary Table 2 | Material parameters in the finite element method (FEM) simulation.
References 1-44.

Supplementary Note 1 | Advantages of piezotronic tunneling junction

Based on piezoelectric theory and band theory, the influence of piezoelectric charges on the current characteristics of Schottky junction (I_{MS}) with metal-semiconductor (MS) contact and the current characteristics of tunneling junction (I_{MIS}) with metal-insulator-semiconductor (MIS) contact can be expressed as follows[1]:

$$I_{MS} = SA^*T^2 exp\left[-\frac{q(\varphi_B + \Delta\varphi_{piezo})}{kT}\right]\left[exp\left(\frac{qV}{nkT}\right) - 1\right] \quad (1)$$

$$I_{MIS} = SA^*T^2 exp\left[-\alpha_T d\sqrt{q\left(\varphi_T + \frac{\Delta\varphi_{piezo}}{2}\right)}\right] exp\left[-\frac{q(\varphi_B + \Delta\varphi_{piezo})}{kT}\right] \left[exp\left(\frac{qV}{nkT}\right) - 1\right] \quad (2)$$

where, S is the contact area, A^* is the effective Richardson constant, q is the amount of electronic charge, k is the Boltzmann constant, and T is the absolute temperature, φ_B is the height of Schottky barrier, $\Delta\varphi_{piezo}$ is the barrier change caused by piezoelectric polarized charges, n is the ideal factor, d is the thickness of insulation layer in tunneling junction, $\alpha_T = 2\sqrt{2qm^*}/\hbar$ ($m^*$is the effective mass of the carrier, $\hbar$ is the reduced Planck constant), φ_T is the effective tunneling junction barrier height.

In comparison with formula (1), the extra term $exp\,[-\alpha_T d\sqrt{q(\varphi_T + \Delta\varphi_{piezo}/2)}]$ in formula (2) indicates that both the tunneling probability of carriers and the barrier height controlled by piezoelectric effect will affect the current characteristics in the tunneling junction. The tunneling probability term $exp(-\alpha_T d\sqrt{q\varphi_T})$ indicates that the carrier tunneling probability is related to the height and width of the tunneling junction barrier.

In the Schottky junctions, the piezoelectric charges only regulate the barrier height. In the tunneling junctions, piezoelectric charges not only regulate the barrier height, but also the tunneling probability of carriers. Thus, compared with the Schottky junction, the tunneling junction has better piezoelectric polarization regulation ability.

Supplementary Note 2 | Material selection

(1) Insulation material selection

There are many literature reports that dielectric insulation layer with high k values and wide band gaps can not only reduce the leakage current and the overall power consumption during device operation, but also has good breakdown resistance reliability in thin film form. As compared with SiO_2, Al_2O_3, MgO and some other insulating materials, HfO_2 has a good advantage in terms of k value and band gap, as shown in **Supplementary Table 1**[2-6]. It should be noted that HfO_2 exhibits good thermal stability and is compatible with silicon technology[2], rendering it a suitable choice for tunneling junctions.

Then, the influence of insulation materials on the performance of piezotronic tunneling junction was studied. Detailly, the strain sensing capabilities of devices with insulation layers of HfO_2, ZrO_2, Al_2O_3 and SiO_2 was evaluated, each with a thickness of ~1.8 nm, under tensile strains of 0.00%, 0.033%, 0.067%, and 0.10%. **Supplementary Fig. 1** presents the current values at a +3.0 V bias for twenty devices of each type, revealing that at 0.00% strain, the currents across the four device types varied randomly between 0.01 and 7 nA, and at 0.10% strain, the variation was between 30 and 250 nA. Analysis of **Supplementary Fig. 1** allows us to calculate and compare the current on/off ratios and the changes in Schottky barrier height for each device type under 0.10% strain, as summarized in **Supplementary Fig. 2**. The results indicate that the insulation materials (HfO_2, ZrO_2, Al_2O_3, and SiO_2) have almost no influence on the strain sensing performance of piezotronic tunneling devices.

In order to understand this phenomenon, we write the classical carrier transport of metal-insulator-semiconductor (MIS) tunneling junction (formula (2) without considering piezotronic effect here)[1]:

$$I = SA^{*}T^{2}exp\left(-\alpha_{T}d\sqrt{q\varphi_{T}}\right)exp\left(-\frac{q\varphi_{B}}{kT}\right)\left[exp\left(\frac{qV}{nkT}\right)-1\right] \quad (3)$$

It can be seen that the tunneling current is mainly dependent on the thickness of insulator layer (d) and the Schottky barrier height (φ_{B}) (which is determined by the metal's work function and the semiconductor's electron affinity), and slightly dependent on the band gap of insulator layer (which together with its electron affinity will affect the effective height of the tunneling barrier $q\varphi_{\mathrm{T}}$), and almost not dependent

on the k value. Given that the band gaps of HfO_2, ZrO_2, Al_2O_3 and SiO_2 are not very different, the piezotronic modification of tunneling current almost doesn't change with the choice of insulation materials, which is consistent with our experimental results in **Supplementary Figs. 1-2**.

Based on the above reasons, HfO_2 was chosen as the insulation layer for convenience, as it can be precisely fabricated using atomic layer deposition (ALD). Actually, other dielectric materials including ZrO_2, Al_2O_3 and SiO_2 can also be candidates as insulation layer.

Supplementary Table 1 | Dielectric constants and band gaps of typical insulation materials [2-6].

Insulation materials	Dielectric constants k	Band gaps E_g (eV)
HfO_2	20~25	5.6~6.0
ZrO_2	17~25	5.1~7.8
SiO_2	3.9	9
Si_3N_4	7~7.5	5~5.3
Al_2O_3	9	8.8
MgO	8.4~9.8	8.7~8.8

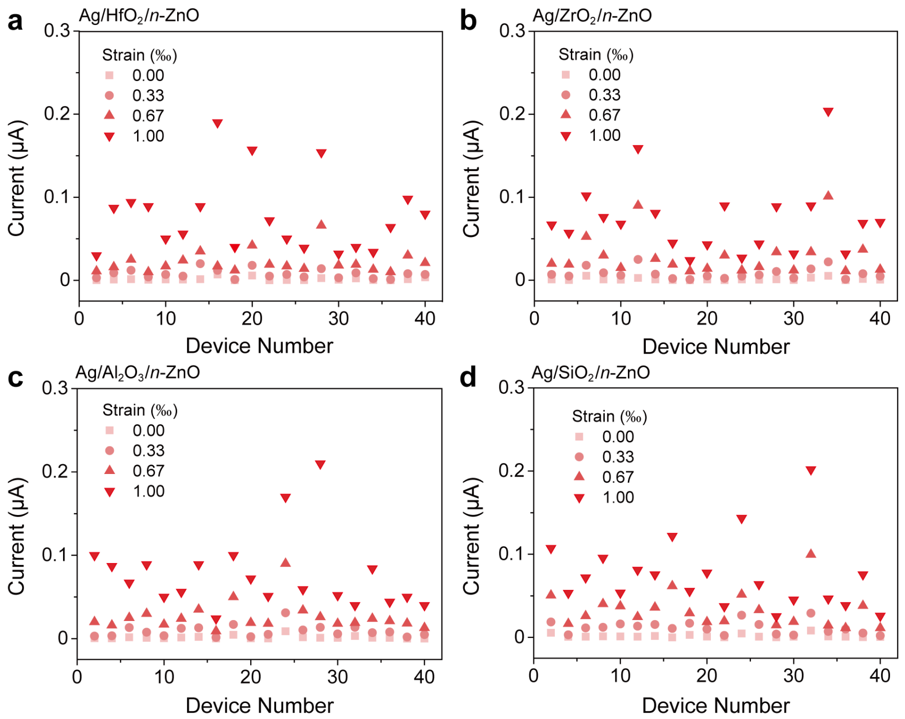

Supplementary Fig. 1 | Influence of insulation materials on piezotronic modification of the carrier transport of piezotronic tunneling devices. a-d, Piezotronic modification of the carrier transports of four types of piezotronic tunneling devices using HfO_2 (**a**), ZrO_2 (**b**), Al_2O_3 (**c**) and SiO_2 (**d**) as insulation layers under the conditions of tensile strain of 0.00%, 0.033%, 0.067% and 0.10%, respectively. The above currents were measured at bias of +3.0 V.

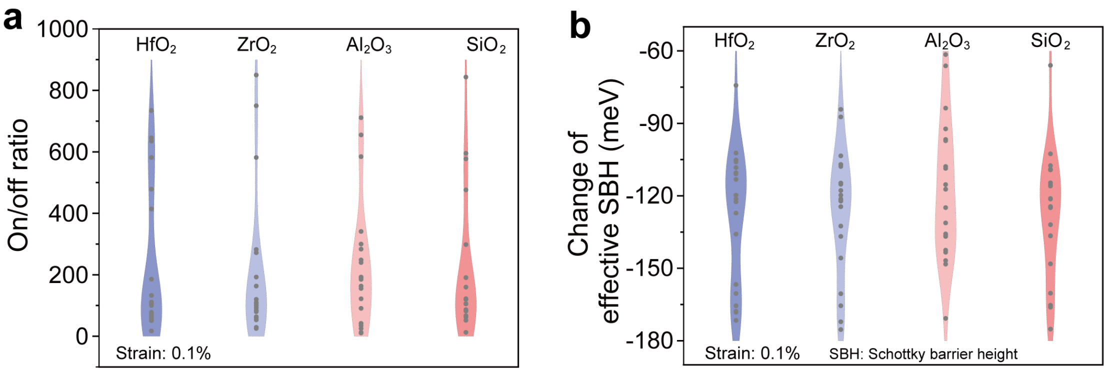

Supplementary Fig. 2 | Sensing performance of piezotronic tunneling devices. a, **b**, On/off ratio (**a**) and change of effective Schottky barrier height (**b**) of the devices based on piezotronic tunneling junction with different insulation materials of HfO_2, ZrO_2, Al_2O_3 and SiO_2.

(2) Selection of thickness for HfO_2 insulation layer

In our experiments, an HfO_2 insulator layer with a thickness of about 1.8 nm was deposited on ZnO nanowire by ALD. We used high-resolution transmission electron microscopy (HTEM) to characterize the thickness of HfO_2 layer. The scanning electron microscopy (SEM) image (**Supplementary Fig. 3a-i**) shows the ZnO nanowire with a hexagonal geometry, and HTEM image of HfO_2/*n*-ZnO (**Supplementary Fig. 3a-ii**) shows a HfO_2 thickness of about 1.82 nm on ZnO surface. In **Supplementary Fig. 3b**, the distribution of HfO_2 thickness along the *x*-axis labeled in **Supplementary Fig. 3a-i** shows that the HfO_2 is uniform with a thickness of about 1.8 (± 0.3) nm. The inset shows the statistical distribution of the insulation thickness.

Here, the rationale for selecting a 1.8 nm thickness for the HfO_2 insulation layer will be briefly elucidated. The Ag/HfO_2/*n*-ZnO devices with HfO_2 thicknesses of 0.0 nm, 0.4 nm, 1.1 nm, 1.8 nm, 2.5 nm, 3.6 nm, and 7.3 nm were fabricated, and then studied the piezotronic modifications of electrical transport of 140 devices (20 devices of each thickness) under 0.10% tensile strain. The experimental results show that the device with 1.8 nm HfO_2 exhibit the highest current on/off ratio (**Supplementary Fig. 4**), which makes us choose 1.8 nm HfO_2 as the insulator layer of piezotronic tunneling junctions.

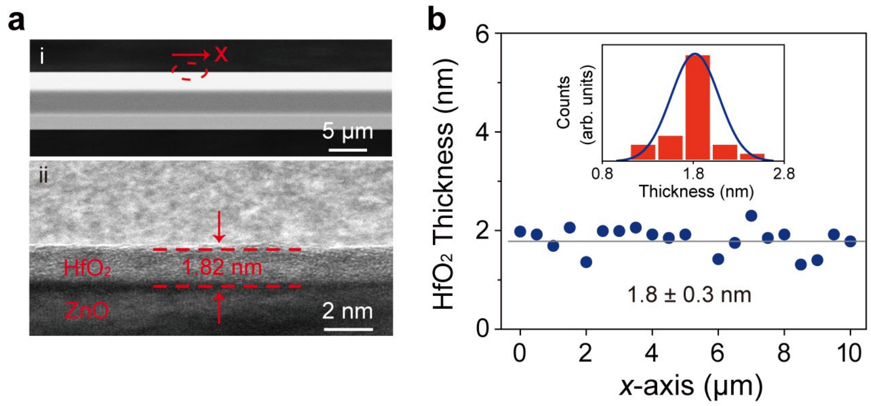

Supplementary Fig. 3 | Morphology of HfO_2/*n*-ZnO microwire and thickness of deposited HfO_2 layer. a-i, SEM image of the ZnO microwire with a diameter of about 8 μm, showing a typical hexagonal geometry and a clean surface. **a-ii**, High-resolution TEM image of HfO_2 layer deposited on ZnO surface, showing a

thickness of 1.82 nm. **b**, Thickness of HfO_2 along the *x*-axis labeled in (**a-i**), showing the HfO_2 thickness of about 1.8 (± 0.3) nm. The inset shows the statistical distribution of the HfO_2 thickness.

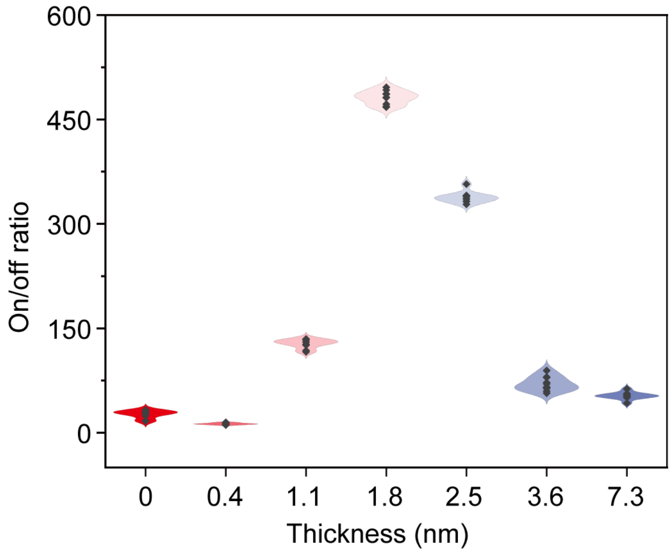

Supplementary Fig. 4 | Current on/off ratio of Ag/HfO_2/*n*-ZnO devices with 0.0 nm, 0.4 nm, 1.1 nm, 1.8 nm, 2.5 nm, 3.6 nm, and 7.3 nm thick HfO_2.

(3) Electrode material selection

There are two reasons for using silver (Ag) as electrodes. Firstly, Ag possesses a relative high work function and can form Schottky contacts with ZnO. As long as the electrode can form a Schottky barrier with ZnO, then the metal-insulation-semiconductor (MIS) tunneling junction can be well constructed. So, Ag meets the requirements. Secondly, in the past ten years, Ag has been widely utilized to fabricate Schottky contacts particularly with ZnO in piezotronic devices[7-9]. Therefore, Ag was chosen as the electrode material for constructing the piezotronic tunneling junction due to its convenience.

(4) Device encapsulation material selection

Generally, pure polydimethylsiloxane (PDMS) is a polymer classified as a silicon elastomer, which usually exhibits no piezoelectric effect. And it is widely used for encapsulating electronics particularly flexible electronics. For example, in the past ten

years, most of piezotronic devices are encapsulated by PDMS. In this work, PDMS is used to encapsulate the piezotronic tunneling junction, protecting it from damage during bending. Pure PDMS exhibits low elastic modulus and strength tunable by curing conditions[10, 11]. Critically, the PET substrate (Young's modulus 3-3.5 GPa) has far greater stiffness than the PDMS film (modulus 360-870 kPa). This enables assuming the nanowire deforms identically to the PET.

We further conducted finite element method simulations under matching experimental conditions to study the possible influence of PDMS encapsulation on performance of the device. Given the micron-scale ZnO nanowire diameter versus millimeter substrate/encapsulation thicknesses, radial nanowire effects are reasonably neglected. Strain loading is modeled by applying proper boundary conditions at the PET substrate neutral layer, inducing bending while preserving neutral layer length. In **Supplementary Fig. 5a**, the FEM model is established with a ZnO nanowire (1 mm in length) that is on the central of PET substrate (0.15 mm in thickness and 30 mm in length). And, a PDMS with thickness 0.5 mm is introduced in a typical model; whereas there is no PDMS structure in the other one for comparison. Here, we list the main parameters of the material properties for the mechanical simulation using the material library database in the software of COMSOL Multiphysics (**Supplementary Table 2**).

It could be clearly observed that the upper part of the piezotronic device is gradually compressed as the increasing curvature of the substrate, as demonstrated by the displacement distribution profiles under different bending conditions in **Supplementary Fig. 5b** and **5c**. **Supplementary Fig. 5d** and **5e** show the corresponding outlines of the bended nanowires obtained from **Supplementary Fig. 5b** and **5c**, while the calculated strain of the ZnO nanowire in the piezotronic device with and without the PDMS layer under the same loading condition is plotted in **Supplementary Fig. 5f**. As can be seen, the existence of PDMS layer would have little influence on the ZnO nanowire deformation when the substrate bends, since the nanowire strain might be identical for the piezotronic device with and without PDMS layer (**Supplementary Fig. 5f**). Therefore, as expected, we chose PDMS for encapsulation of devices.

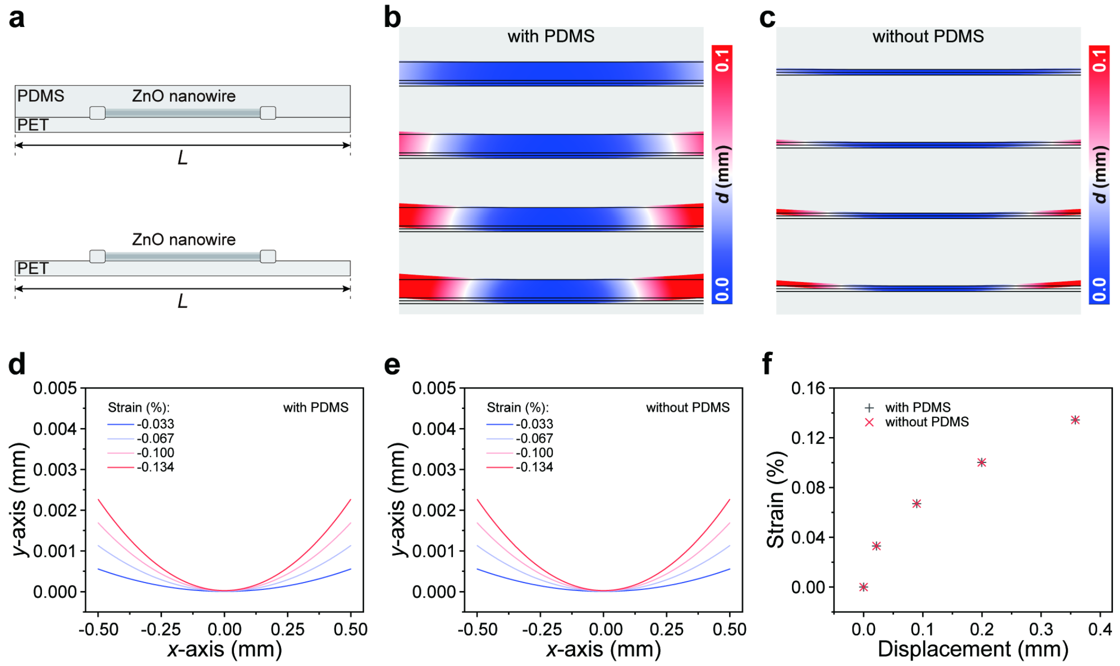

Supplementary Fig. 5 | Impact of the PDMS encapsulation layer on the performance of the piezotronic tunneling device by FEM simulation. a, Schematic illustration of the side view of the piezotronic tunneling device with (top) and without (bottom) PDMS encapsulation layer. **b-e**, Displacement distribution profiles (**b**, **c**) and the corresponding outlines of bended nano/microwires (**d**, **e**) of the piezotronic tunneling devices with (**b**, **d**) and without (**c**, **e**) PDMS layer in response to mechanical strain, respectively. **f**, Calculated ZnO nano/microwire strain as a function of the distance change between the two ends of the substrate in the piezotronic device with and without the PDMS layer, respectively.

Supplementary Table 2 | Material parameters in finite element method simulation.

Materials	Young's modulus, *E*	Poisson's ratio, *v*
PET	4 GPa	0.35
PDMS	750 kPa	0.49
ZnO	210 GPa	0.33

Supplementary Note 3 | Material characterization

In this work, the one-dimensional ZnO nano/microwires synthesized by chemical vapor

deposition (CVD) method are used to prepare the piezotronic tunneling junctions[12]. After the preparation of ZnO nano/microwires, we use scanning electron microscopy (SEM), transmission electron microscopy (TEM) and X-ray diffraction spectroscopy (XRD) to characterize the morphology, high-resolution lattice characteristics and crystal quality of the materials, respectively. The SEM image in **Supplementary Fig. 6a** shows that the diameter of ZnO nano/microwire is about 8 μm. It can be clearly observed from the figure that the prepared nano/microwire has regular hexagonal geometry with the clean and flat surface. The TEM image in **Supplementary Fig. 6** shows the surface lattice images of a single ZnO microwire at different positions with high magnification. The clear boundary of the high-resolution surface and clear lattice fringe in each inset indicate that ZnO nano/microwire has smooth surface and good crystallinity. **Supplementary Fig. 6c** shows the X-ray diffraction spectra of ZnO nano/microwires. The characteristic peaks measured in XRD patterns correspond to the completely different diffraction peaks observed on different crystal planes of ZnO. This further proves that the prepared ZnO piezoelectric materials have good crystallization properties.

In order to obtain the cross-section image of the tunneling junction, we used a small tool knife to cut the end of the ZnO microwires deposited by HfO_2 and Ag. **Supplementary Fig. 7a** shows the high-resolution SEM image of the Ag/HfO_2/*n*-ZnO junction, revealing the hexagonal cross-section of ZnO and the Ag electrode coating. Additionally, **Supplementary Fig. 7b** presents an enlarged cross-section image of the junction. The thickness differences between the HfO_2 insulation layer (only 1.8 nm) and the Ag electrode make it difficult to clearly observe the HfO_2 layer on the nanowire surface. Following this, we conducted an EDX elemental mapping analysis based on the high-resolution cross-section image in **Supplementary Fig. 7c**. The EDX element mappings for Zn, O, Hf, and Ag in **Supplementary Fig. 7d**, allowing us to observe the distribution of the Hf element at the boundary between Ag and ZnO.

Defects in HfO_2 can introduce defective surface, which may make a big influence on the tunneling junctions. In order to verify the influence of possible defective surfaces, we characterized the surface of ZnO and conducted capacitance measurements on

Ag/HfO_2/*n*-ZnO tunneling junctions to assess the interface traps formed in the tunneling junction, thereby demonstrating the impact of defects in HfO_2 layer.

In addition, capacitance measurements have been used to quickly assess the impact of interface traps on the tunneling properties of metal-insulator-semiconductor (MIS) tunneling junctions[1]. Here, we conducted *C-V* characterizations on 40 fabricated devices, as illustrated in **Supplementary Fig. 8**. The results displayed two typical *C-V* curves of Ag/HfO_2/*n*-ZnO tunneling junctions (**Supplementary Fig. 8a**). According to the research and theories of Tamm[13], Shockley[14, 15], and others regarding interface traps [16] (also historically referred to as interface states, interface defects, surface states, and so on), it is evident that interface traps significantly influence the *C-V* curve, causing it to elongate in the voltage direction[1]. This is attributed to the additional charges required to fill the traps, resulting in a greater total charge or applied voltage needed to achieve the same surface potential or band bending. Furthermore, interface traps also impact the total capacitance of the tunneling junction. With a fixed bias, the presence of interface traps reduces the remaining charge available to be placed in the depletion layer, thereby diminishing the band bending or surface potential. As a result, the fundamental characteristics of the *C-V* curve with interface traps shift in both the voltage and capacitance directions, leading to a wider curve opening. Consequently, the blue curve in **Supplementary Fig. 8a** closely resembles the ideal *C-V* characteristics of an MIS tunneling junction with few interface traps[1], while the red curve, elongated in the voltage direction, represents a device with noticeable interface traps. **Supplementary Fig. 8b** presents the characterization results of the 40 fabricated devices, revealing that only a small percentage (~3.85%) exhibited obvious interface traps.

The process of atomic layer deposition (ALD) of HfO_2 is a self-limiting growth process, with each cycle depositing only one atomic layer or a few atomic layers. Once the surface is occupied by precursor molecules, no further reactions occur, indicating that the growth of the thin film is highly controllable, thus reducing the generation of defects. In this study, the 1.8 nm-thick HfO_2 film deposited by atomic layer deposition is extremely thin, approaching the thickness of a monolayer or a few atomic layers. For the 1.8 nm-thick HfO_2 film, we have rigorously controlled and optimized all steps of

the ALD process and used high-purity precursors, therefore, the grown HfO_2 film should have a lower defect density.

Combining the surface quality characterization of ZnO nanowires and the *C-V* characteristics of devices, we think the influence of interface traps (including interface defects in ZnO and HfO_2) on Ag/HfO_2/*n*-ZnO can be neglected.

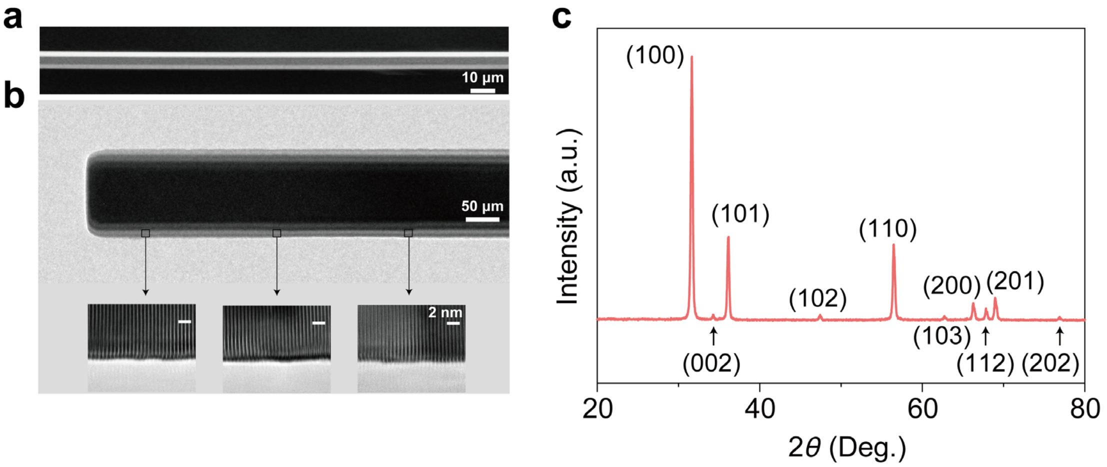

Supplementary Fig. 6 | Scanning electron microscopy (SEM), transmission electron microscopy (TEM) images, and X-ray diffraction spectra of ZnO microwire. a, **b**, SEM (**a**) and TEM (**b**) images of ZnO microwire used in this work. The insets in (**b**) exhibit the surface quality of the ZnO microwire at different locations labeled in black box. The scale bars of insets represent 2 nm. **c**, Corresponding peaks of (100), (002), (101), (102), (110), (112) and (201) planes indicate that the ZnO microwires were highly crystalline in nature.

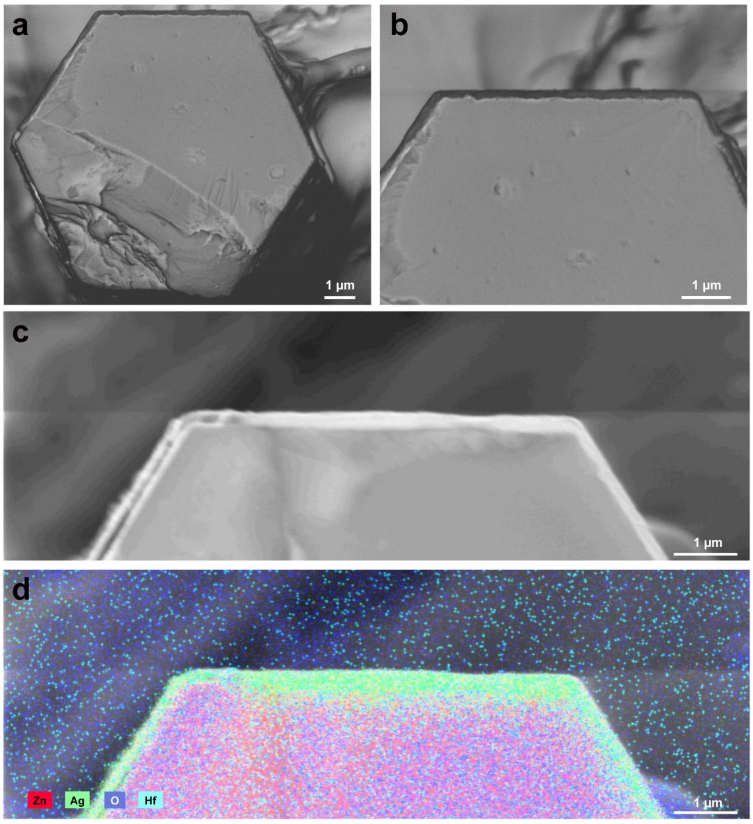

Supplementary Fig. 7 | Crosse-section TEM and SEM images of Ag/HfO_2/*n*-ZnO junctions and the corresponding EDX element mapping image. a, Crosse-section view SEM images of an Ag/HfO_2/*n*-ZnO junction. **b**, Enlarged version of the cross-section SEM image of the Ag/HfO_2/*n*-ZnO junction in (**a**). **c**, High-resolution cross-section view SEM image of the Ag/HfO_2/*n*-ZnO junction. **d**, The corresponding EDX element mapping image of Zn, Ag, O and Hf in (**c**).

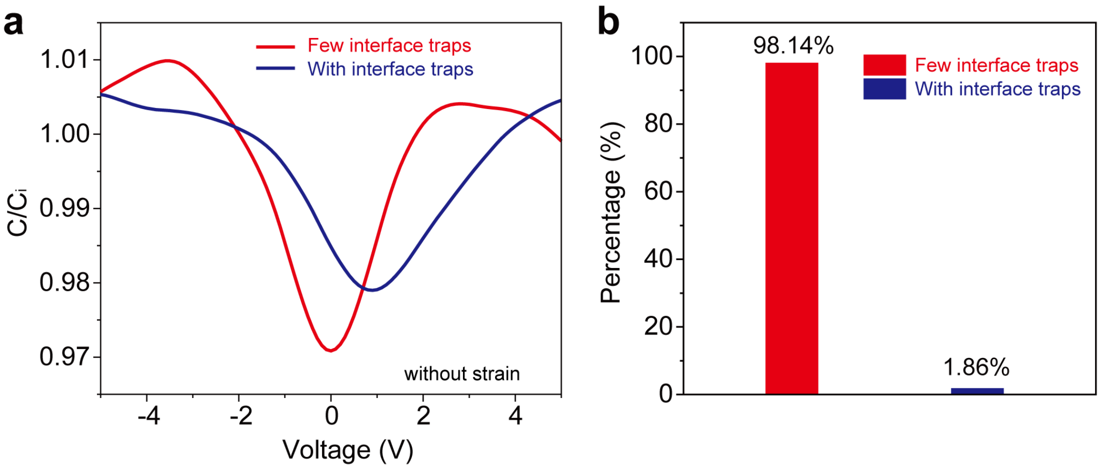

Supplementary Fig. 8 | Influence of interface traps on *C-V* curves of Ag/HfO_2/*n*-

ZnO tunneling junctions. a, Two typical *C-V* curves of Ag/HfO_2/*n*-ZnO devices under strain free condition. The red curve is close to the ideal *C-V* characteristics of Ag/HfO_2/*n*-ZnO tunneling junction. The blue curve is stretched out in the voltage direction, which represent the device with obvious interface traps. **b**, Percentages of 40 devices with few interface traps and obvious interface traps occurred in experiments.

Supplementary Note 4 | Piezoelectricity in wurtzite ZnO

The ZnO nano/microwire has wurtzite crystal structure and its physical properties are coupling of piezoelectric properties and semiconductor properties. Because the (0001) surface of ZnO is terminated by zinc and oxygen atoms during the growth process, the entire crystal structure lacks central symmetry. As shown in **Supplementary Fig. 9a**, when the strain is applied, the piezoelectric charges will be generated on the two surfaces of the crystal along the *c*-axis, which is macroscopically manifested as the polarization of ZnO along its (0001) plane, resulting in a macroscopic piezoelectric potential. The characteristics of piezoelectric charges can be controlled by changing the magnitude and direction of strain. According to the piezoelectric theory, when the applied force is consistent with the direction of the polarized *c*-axis, the end face along the direction of the *c*-axis will generates positive piezoelectric charges, while the end face against the direction of the *c*-axis will generate negative piezoelectric charges. When the direction of the force is changed, the polarity of the piezoelectric polarization charges at both ends of the nano/microwire also changes.

For piezoelectric semiconductor materials, the piezoelectric constant determines the amount of piezoelectric charges induced by pressure, and therefore plays a key role in the performance of the piezoelectric semiconductor device. The characterization technique based on atomic force microscopy (AFM) can measure the relationship between piezoelectric properties and mechanical forces of nanomaterials on the atomic scale. Here, we use the piezoelectric response force mode (PFM) of AFM to study the piezoelectricity of ZnO nano/microwires. Most researches on piezoelectric properties of materials are based on positive or inverse piezoelectric effect. The principle of characterization measurement under PFM mode is according to the inverse

piezoelectric effect of semiconductor materials. When an AC electrical signal is applied to the probe, the material will undergo tensile or compressive deformation under the action of alternating electric field. This deformation signal will be detected by the working equipment in PFM mode, and the value of the piezoelectric characteristics of the measured material will be calculated automatically by the system software. It is worth noting that the slope of the curve of the amplitude in relation to the applied voltage measured under PFM mode represents the piezoelectric coefficient (d_{33}). As shown in **Supplementary Fig. 9b**, obvious amplitude changes can be observed as the tip voltage increases, and the slope of the measured amplitude-voltage curve indicates that the piezoelectric constant d_{33} of ZnO nano/microwires is about 11 pm V^{-1}. Compared with some piezoelectric materials[17-23], this relatively high piezoelectric coefficient value also proves that the ZnO nano/microwires prepared by CVD method has good piezoelectric properties, which are more suitable for the study of piezotronic devices.

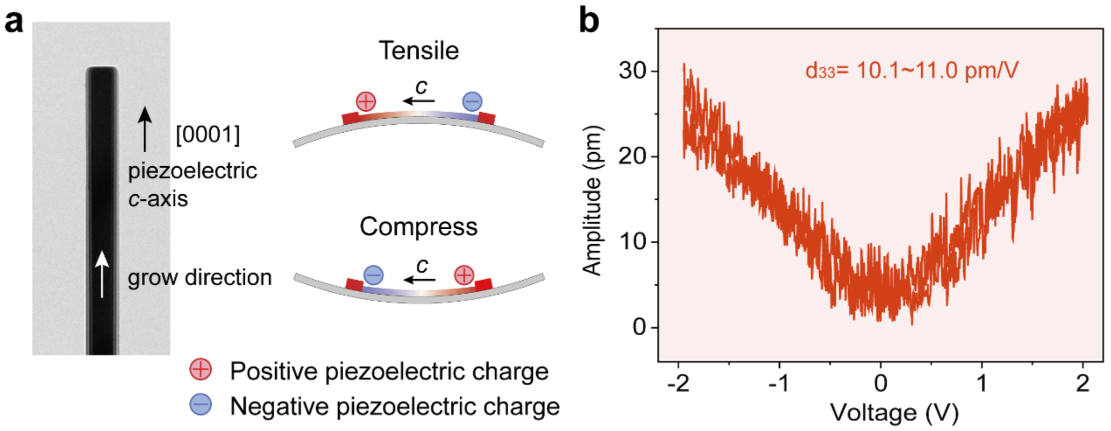

Supplementary Fig. 9 | Piezoelectricity in wurtzite ZnO. a, Transmission electron microscopy images of ZnO microwire (left) and schematic diagram of piezoelectric charge generated under strain in ZnO nano/microwires. The white arrow and the black arrow are the growth direction and the piezoelectric polarization axis direction of the microwire, respectively. In the schematic diagram of the ZnO nanowire, the red and blue colors represent positive and negative potentials, respectively. **b,** Piezoelectric coefficient (d_{33}) of ZnO nano/microwires.

Supplementary Note 5 | Piezoelectric nanogenerator measurement to determine the polarity of ZnO nano/microwires

The ZnO nano/microwires used in the experiment are oriented along the *c*-axis. However, there are many uncertainties in the actual experiment process, which will result in the unknown *c*-axis direction of piezoelectric materials. When the *c*-axis direction of the nano/microwire is uncertain, we can judge it according to the output signal of the piezoelectric nanogenerator (PENG). As for the PENG, once the nano/microwires are subjected to a mechanical deformation, the piezoelectric potential resulted by the piezoelectric charges can be used as the driving force for electron flow in the external load. **Supplementary Fig. 10a** shows the current response of PENG based on ZnO nano/microwire under different compressive strains. When the PENG is stimulated by mechanical force, the negative peak current signal can be got first, and when the mechanical force is released, a positive peak current signal will be observed. The working mechanism diagram of the PENG in **Supplementary Fig. 10b** can be used to explain the phenomenon of the data in **Supplementary Fig. 10a** and determine the direction of the *c*-axis of ZnO nano/microwires. As shown in **Supplementary Fig. 10b-i**, when no strain occurs in ZnO nano/microwire, no current passes through the test circuit. When ZnO nano/microwire is subjected to an external mechanical force of compression (**Supplementary Fig. 10b-ii**), driven by the piezoelectric potential generated by piezoelectric polarization charge, a right-to-left reverse current signal will be generated in the current loop of the measurement system. When the compression stress is released (**Supplementary Fig. 10b-iii**), the deformation of the device is restored, and a left-to-right positive current signal will be also generated in the current loop. This proves that under the state of compressive strain, the right end of the nano/microwire generates positive piezoelectric polarization charges, while the left end generates negative piezoelectric polarization charges. Therefore, it can be determined that the direction of the piezoelectric *c*-axis of this ZnO nano/microwire is oriented from the right to the left. The strain-induced piezoelectric potential is the basis of the generation of PENG. When the direction of the mechanical force applied to the device is changed, the polarity of the piezoelectric charges generated at both ends of the nano/microwires will also change,

causing a shift in the polarity of the output peak current by the PENG. It should be noted here that the piezoelectric charges generated on the (0001) plane of ZnO nano/microwire play a leading role in this work.

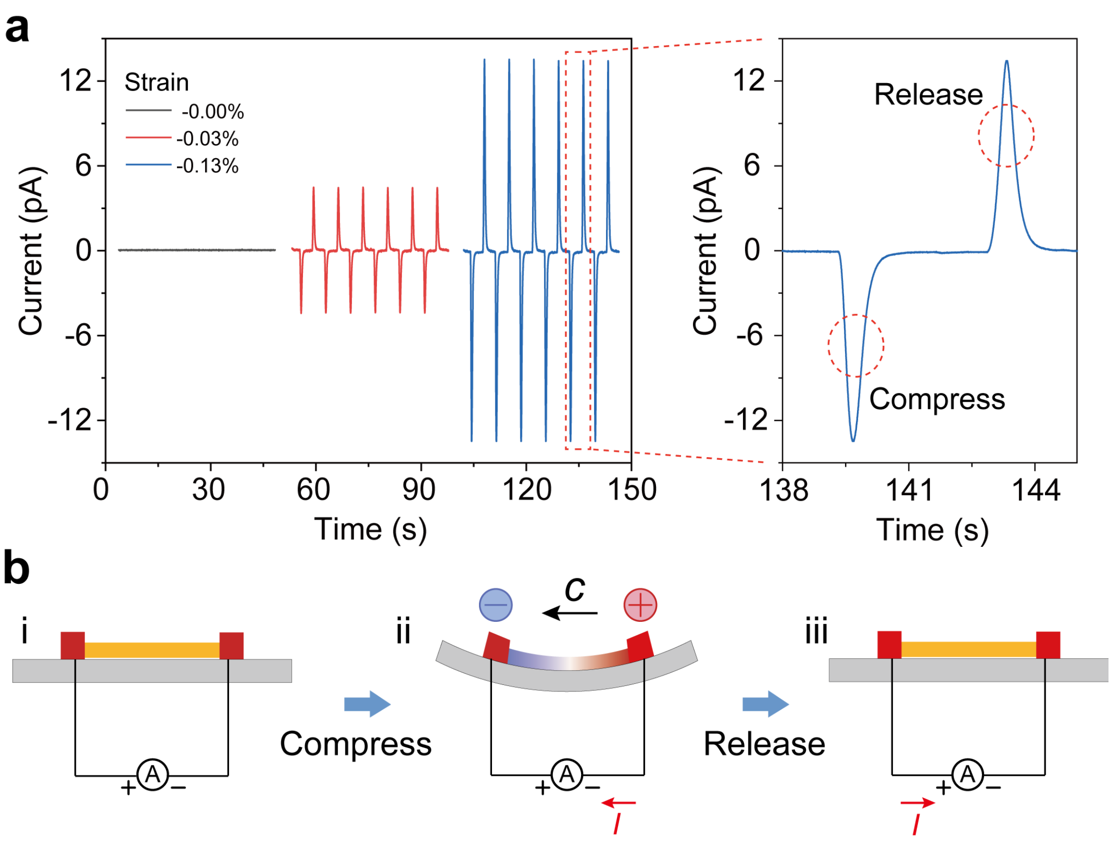

Supplementary Fig. 10 | Piezoelectric nanogenerator (PENG) measurement to determine the *c*-axis orientation of the ZnO nano/microwire. a, Current output of PENG during the compress-release cycles to determine the *c*-axis direction of the ZnO nano/microwire. **b,** Schematic diagrams of working mechanism for the PENG based on a single nano/microwire upon compressive strain.

Supplementary Note 6 | Effect of piezoelectric polarization on the barrier width of tunneling junction

In this work, piezotronic tunneling junction consists of insulating barrier and Schottky barrier. Based on the band theory, we will discuss the influence of strain on the energy band distribution of Ag/HfO_2/*n*-ZnO tunneling junction in **Supplementary Fig. 11**. In the strain-free thermal equilibrium state, a wide depletion layer is formed on the *n*-ZnO side of the Ag/HfO_2/*n*-ZnO contact interface (yellow area in **Supplementary Fig. 11a**). The free electrons on the conduction band of ZnO need high energy to pass through the barrier with a certain width, so only a few carriers can pass through the tunneling junction area. At this time, the tunneling junction is in the off state of high resistance. The red arrow in the figure indicates the electron transport characters. When *n*-ZnO is stressed along the *c*-axis direction, the positive piezoelectric charges caused by compressive strain will be generated at the interface of HfO_2/*n*-ZnO and bend the interface energy band downward (**Supplementary Fig. 11b**).

In this case, the width of the depletion layer on *n*-ZnO side will become narrower, and electrons on the conduction band can pass through the lowered barrier with less energy, so large numbers of electrons will tunnel (**Supplementary Fig. 11b**). At this time, piezotronic tunneling junction is in the on state of low resistance, and the carrier transport characteristics can be changed by regulating the barrier width.

Next, we use WKB approximation to simulate the potential distribution of Ag/HfO_2/*n*-ZnO tunneling junction under strain regulation[1]. In **Supplementary Fig. 12**, with the increase of strain, the voltage drop will gradually transfer from semiconductor (*n*-ZnO) to metal (Ag), and gradually concentrate on the insulating layer (HfO_2), indicating that the effective width of the barrier at the semiconductor side is decreasing. Thus, the strain induced positive piezoelectric charge will reduce the effective barrier width of the tunneling junction, which is consistent with the band diagram analysis in **Supplementary Fig. 11**.

Through the above theoretical and simulation analysis, we can confirm that the strain induced piezoelectric charge can tune the effective barrier width of the tunneling junction (Ag/HfO_2/*n*-ZnO).

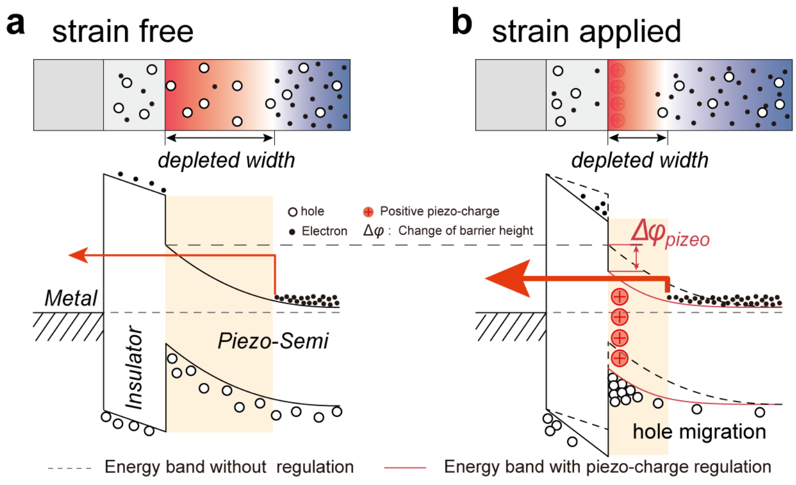

Supplementary Fig. 11 | Barrier profiles of the Ag/HfO_2/*n*-ZnO piezotronic tunneling junction under strain-free (a) and strain (b). The shadow represents the depletion layer width. In the schematic diagram of *n*-ZnO, the red and blue colors represent positive and negative potentials, respectively.

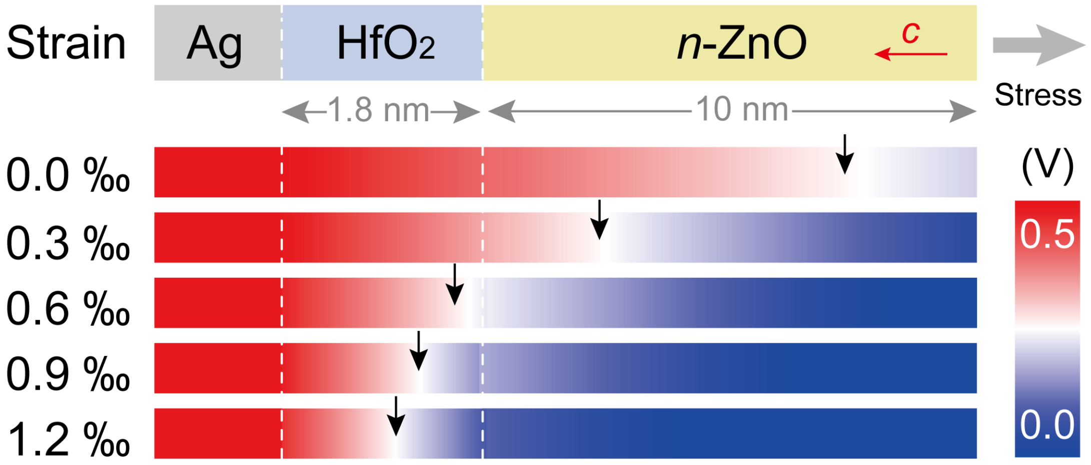

Supplementary Fig. 12 | Calculated potential distribution of Ag/HfO_2/*n*-ZnO tunneling junction under different strain ranged from 0.0 to 1.2 ‰ along *c*-axis.

Supplementary Note 7 | Novelty of piezotronic tunneling junction with tunable interface barrier in this work

Piezotronic devices usually possess high sensitivity to response to external mechanical stimuli in theory. As shown in **Supplementary Fig. 13**, the basic structure of piezotronic devices is mainly composed of piezoelectric semiconductor and electrodes at both ends. The electrical transport of the device is dependent on the interface barrier and highly sensitive to the interface barrier height. The strain-induced piezoelectric polarization charges and corresponding piezo-potentials at interfaces are able to linearly modulate the interface barrier height, and hence exponentially control the electrical transport across the interface[24, 25]. Because of this natural exponential relationship between input mechanical signal and output electrical signal, piezotronic device has inherent advantages in detecting mechanical stimuli. However, there is a disadvantage for piezotronic devices that once they are fabricated, the internal interface barrier is fixed, leading to the inability to tune the device performance. This means that the range of response current for the same strain will also be fixed, which may render piezotronic devices unsuitable for certain applications. Since polarized interface engineering highly depends on the nature of the interface barrier, once the interface potential barrier is established, its fixed value and uncontrollability are not beneficial to expand the performance and application scenarios of polarization interface engineering.

If the interface barrier of piezotronic tunneling junction can be tuned, the tunable piezotronic devices can be realized for more scenarios and wider applications. Besides, it may also inspire the researches on tunable electronic devices based on some other interface engineering by local polarizations like flexoelectric, and ferroelectric polarization (flexoelectronic devices and ferroelectric electronic devices)[1, 8, 26]. Therefore, developing the strategy for piezotronic tunneling junction with tunable interface barrier is important.

As presented in the manuscript (Figure 3), a strategy of tuning piezotronic effect reversibly and accurately by electric pulse is achieved in the constructed Ag/HfO_2/*n*-ZnO piezotronic tunneling junction. Considering the obvious regulation effect of

electric pulse on the interface barrier in piezotronics devices, our work conquered the current disadvantage of fixed interface barrier in piezotronics.

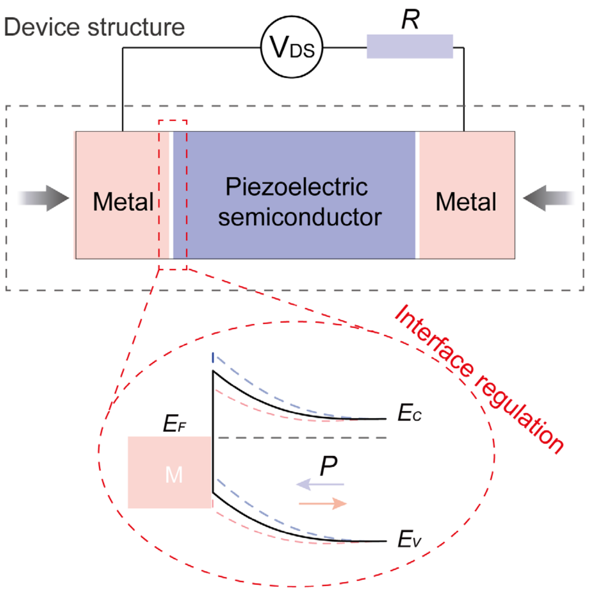

Supplementary Fig. 13 | The basic typical structure of piezotronic devices.

Supplementary Note 8 | Experimental setup and electrical measurement

The measuring equipment used in the experiment mainly includes low noise preamplifier (SR570, SR560), Keithley2636b digital source meter and DC linear motor. PCI-6259 (National Instrument) is used for test data acquisition. The strain of the device is mainly driven by the linear motor. Low noise preamplifier (SR570, SR560) equipment can accurately measure and display the output voltage and current of the device under strain state. Keithley2636b digital source meter can be used for input electric pulse voltage signal, as well as output pulse current response signal. **Supplementary Fig. 14** shows the circuit connection diagram used in the working process of the device with piezotronic tunneling junction. The diagram shows that the Keithley2636b digital source meter and the electrical measurement system can be independently connected to the two electrical extremes of the device, forming a complete current loop. In the characterization of electrical properties, we first let the pulse voltage set by the program stimulate the device for a certain time, and then

connect the two electrical extremes of the device to the electrical measurement system for current-voltage (*I-V*) characteristics test.

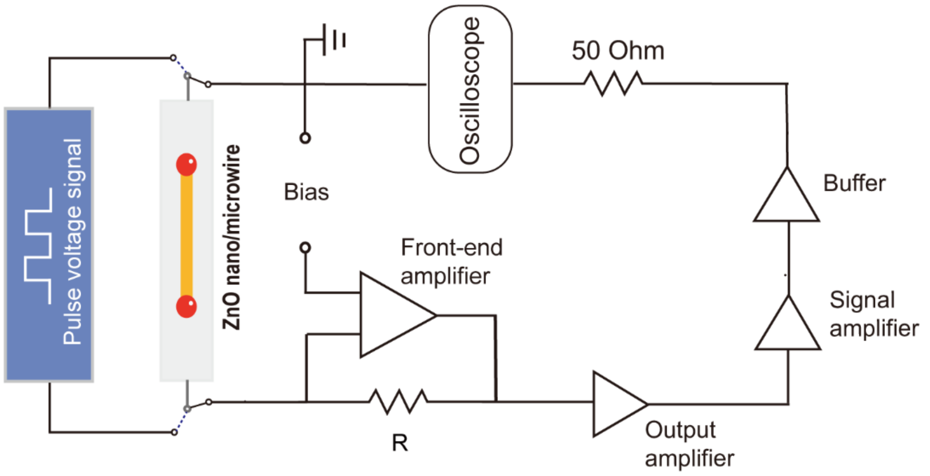

Supplementary Fig. 14 | Schematic diagram for the experimental setup of the electrical measurement system.

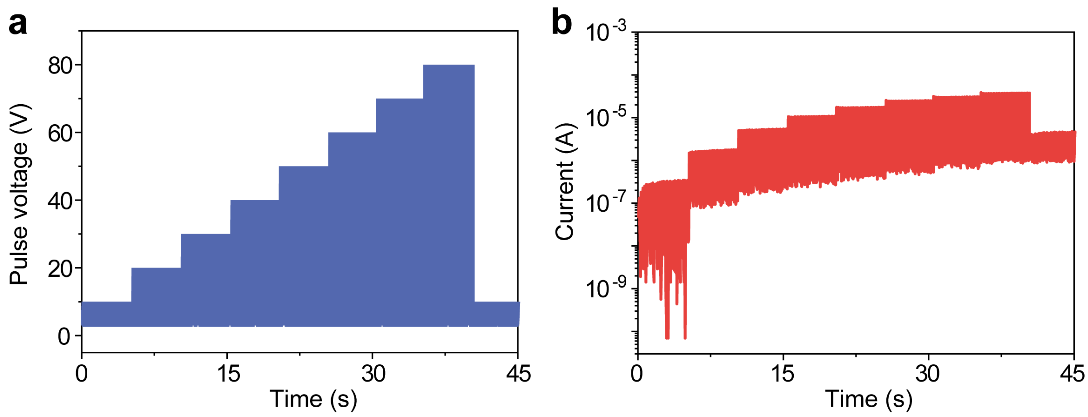

Supplementary Fig. 15 | Electric pulse stimulation and current response of the device with tunneling junctions. a, Amplitude of the electric pulse voltage stimulating the device is 10 V, 20 V, 40 V, 60 V and 80 V, respectively. The time interval between electric pulses is 0.1 s, and the pulse duration is 0.05 s. **b,** Current response corresponding to (**a**).

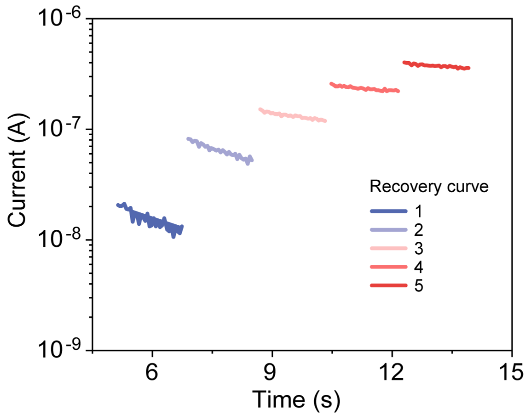

Supplementary Fig. 16 | Five recovery curves of the device with tunneling junctions under electric pulse stimulation.

Supplementary Note 9 | Potential methods to achieve a long retention of the tunable barrier height

There are three potential methods to increasing the retention of tunable barrier height:

(1) Appropriately reducing the period time or increasing the duration of electric pulse

By reducing the period time of electric pulses, sufficient electric force and energy per unit time can be introduced to drive vacancy migration and redistribution, and increase the response current, leading to a higher change of the barrier height. After the stimulation of electric pulse with small period time, the vacancies require more time to recover to their origin place/state. As a result, a long retention of the tunable barrier height can be achieved by this method.

Increasing the duration of electric pulse is also an effective way to achieve a long retention of tunable barrier height. As shown in **Supplementary Fig. 17**, electric pulse with a same pulse voltage of 40 V but longer duration will lead to a larger response current of the device (**Supplementary Fig. 17b-c**) and a smaller recovery rate of the current after stimulation (**Supplementary Fig. 17d**). In other words, after the stimulation of electric with long duration, the vacancies require more time to recover to their origin place/state. It is important to note that increasing the duration of electric pulse may also have some negative influences, such as increased power consumption, thermal effect, and negative impacts on the long-term stability of the device.

Thereby, appropriately reducing the period time or increasing the duration of electric pulse can achieve a long retention of the tunable barrier height.

(2) Appropriately annealing the semiconductors

Appropriately annealing may adjust the internal structure and properties of the material, making it possible to achieve a long retention of the tunable barrier height. Annealing of the *n*-ZnO nanowire in air can increase the density of vacancy and slightly increase the recovery time, indicating annealing is a possible method to achieve long retention. Besides, literatures[27-29] also give some other reasons to use annealing to improve the retention. ① Crystal defect repair: during annealing, high temperatures can promote the migration and binding of lattice defects, thereby reducing or repairing crystal defects within the material. This helps improve the crystalline quality and grain boundary conditions, stabilizing the adjustability of the barrier height. ② Lattice stress release: proper annealing can help release lattice stress within the material, improving the crystal structure and stability, thus affecting the long-term maintenance capability. ③ Interface barrier adjustment: during annealing, the interface properties between semiconductor materials and metals or other semiconductor materials can be adjusted, affecting the quality of the barrier formation. This contributes to the long-term stability of the barrier height. Thereby, appropriately annealing can improve the structure and properties of semiconductors, possessing possibility to achieve a long retention of the tunable barrier height.

(3) Continuously applying a small electric pulse

After the stimulation of electric pulse with high amplitude, continuously applying a small electric pulse (different from the former electric pulse with high amplitude) together with the sweeping voltage (used to measure the *I*-*V* characteristics) may maintain the vacancy redistribution. In theory, the form of small electric pulse can be easily distinguished from the sweeping voltage, thereby the measured current containing a DC part and a pulse part can be also distinguished. As a result, the applied small electric pulse to maintain the vacancy redistribution and the tunable interface barrier height will not affect the *I*-*V* characteristics of the device. This method should be feasible in theory, but a large number of experiments are needed to find the

appropriate amplitude, frequency and duration of the small electric pulse, which needs further experimental demonstration in the future.

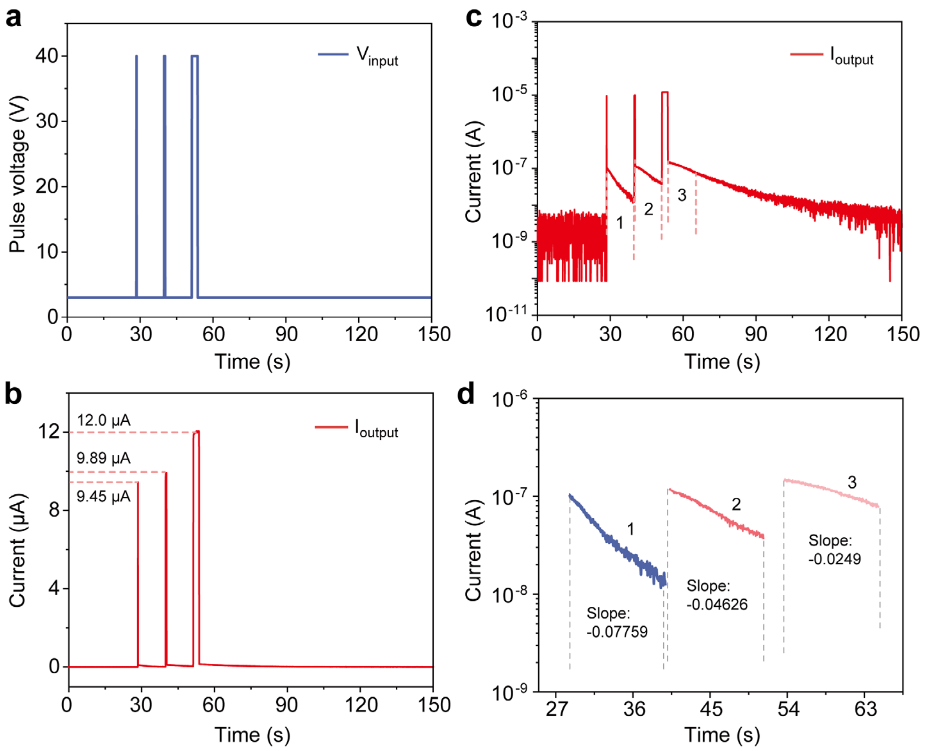

Supplementary Fig. 17 | Current response and recovery characteristics regulated by the electric pulse duration. a, Electric pulse voltage of 40 V on Ag/HfO_2/*n*-ZnO device with the duration of each pulse of 0.05 s, 0.36 s and 2.35 s, respectively, and the time interval between two electric pulses is about 10.96 s. **b**, **c**, Current response corresponding to (**a**). **d**, Three current recovery curves numbered '1' to '3' in (**c**), in which the recovery slopes are -0.07759, -0.04626 and -0.0249, respectively.

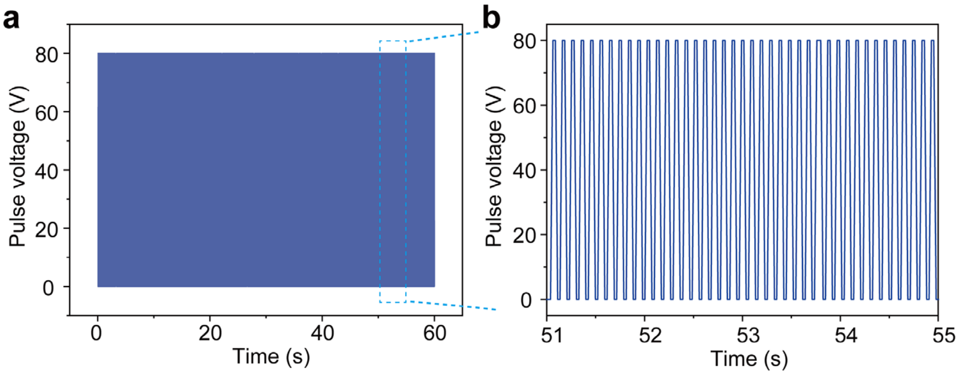

Supplementary Fig. 18 | Electric pulse voltage used to stimulate the device. a, 80 V

electric pulse voltage lasting for 60 s. **b,** Enlarged view in the box in (**a**). The frequency of electric pulses is 10 Hz.

Supplementary Note 10 | Selection and impact of pulsed electric fields (intensity, frequency, etc.)

(1) Pulsed electric field

① Pulse amplitude: The electrical characteristics of device were evaluated under the periodic electric pulse stimulation with a frequency of 10 Hz and amplitudes of 70 V, 80 V, 100 V, 120 V and 150 V, respectively. **Supplementary Fig. 19** shows the electric pulse with various amplitudes (left side) applied on the device and their corresponding current responses (right side). As can be seen from **Supplementary Fig. 19a-d**, the current response to the pulses with amplitude from 70 V to 120 V all increase within 60 s (**Supplementary Fig. 19a-1** to **19d-1**). However, when the amplitude of the pulse is 150 V (**Supplementary Fig. 19e**), the current response increases to 118 μA within 0.3 s, but then shows a decaying trend. And, as the periodic pulse voltage lasts for 60 s, the current response downs to 71.4 μA. This indicates that the pulse amplitudes under 120 V do not damage the device, but 150 V is high enough to cause device breakdown.

Supplementary Fig. 20a shows the *I-V* characteristics after 60 s electric pulse with amplitudes of 0 V, 70 V, 80 V, 100 V, 120 V and 150 V in **Supplementary Fig. 19a-d**. Consistent with the phenomenon shown in **Supplementary Fig. 19**, the electrical transport of the device continue to increase as the pulse amplitude increases from 0 V to 120 V, but attenuates after the pulse of 150 V (**Supplementary Fig. 20a**). **Supplementary Fig. 20b** summarizes the current value at +1 V after the electric pulse with various amplitudes from **Supplementary Fig. 20a**. It can be observed that the current value drops significantly as the pulse amplitude reaching 150 V, which also means that 150 V is high enough to cause device breakdown.

So, the devices can withstand electric pulse with amplitude under 120 V (60 s at 10 Hz), and the 80 V electric pulse used in this work will not damage the devices.

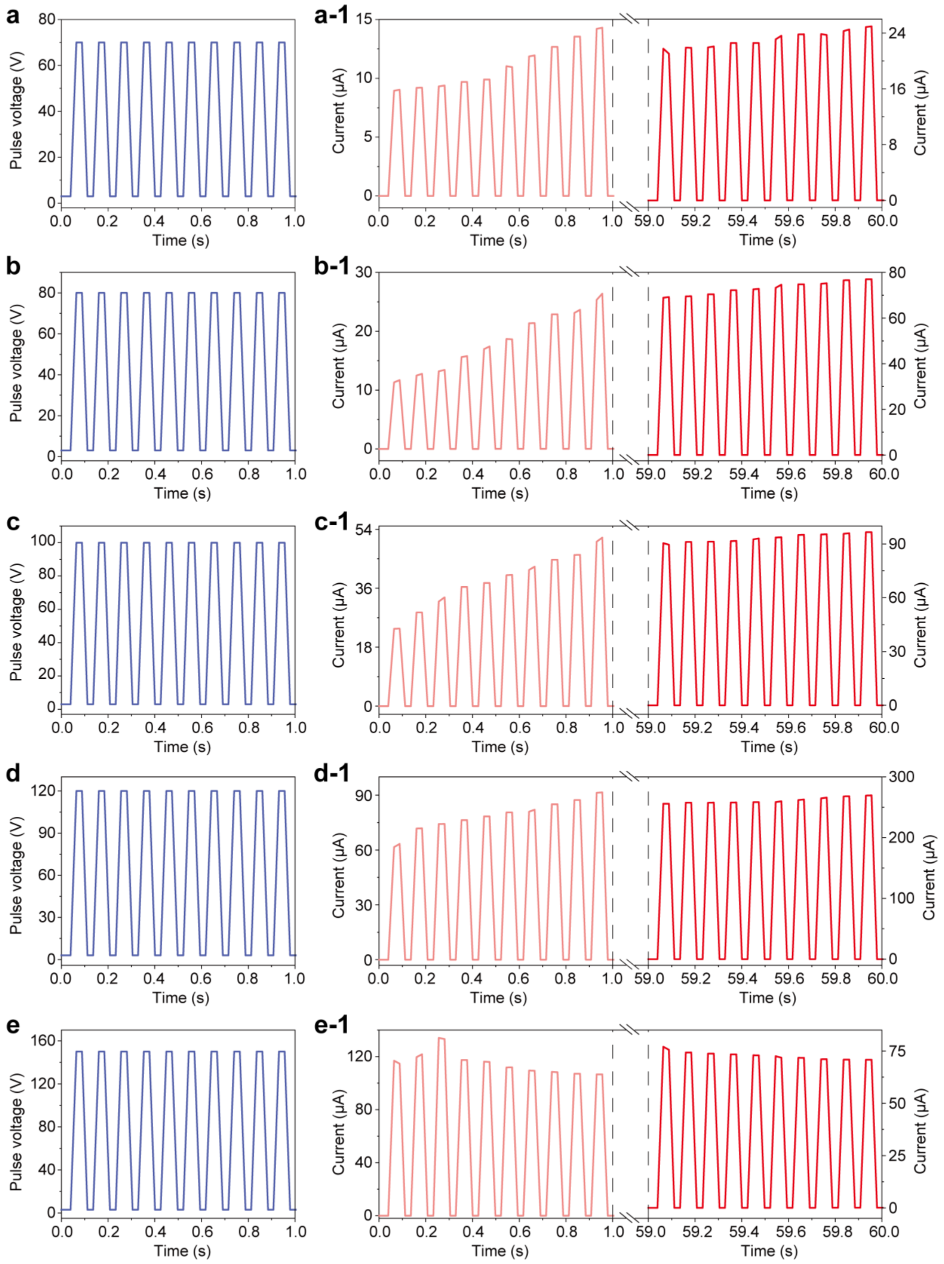

Supplementary Fig. 19 | Electric pulse voltage and the corresponding current of the device. a-e, Amplitude of electric pulse stimulating the device is 70 V, 80 V, 100 V, 120 V and 150 V, respectively. Both of the time interval between electric pulses and pulse duration are 0.05 s. (**a-1**)-(**e-1**), Current corresponding to (**a-e**) within 60 s.

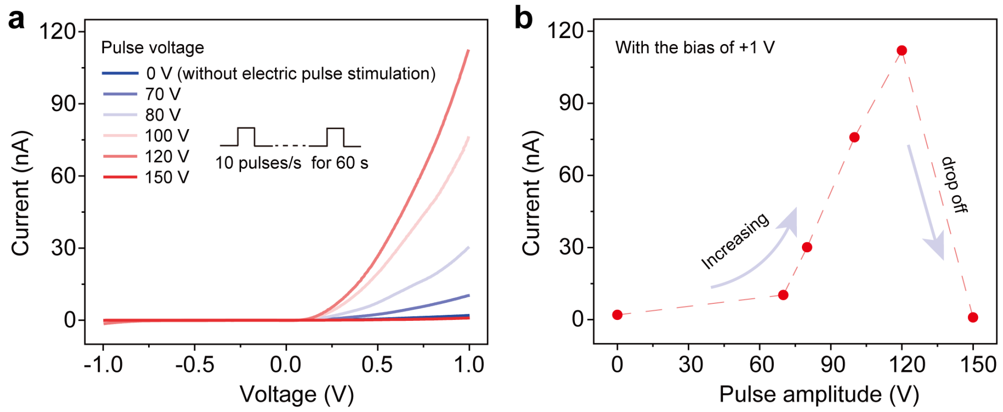

Supplementary Fig. 20 | *I-V* characteristics after the electric pulse stimulation. a, *I-V* characteristics after the electric pulse stimulation for 60 s with different pulse amplitudes of 0 V, 70 V, 80 V, 100 V, 120 V and 150 V, respectively. **b**, Current value at +3 V obtained from **a**. When the pulse voltage amplitude increases from 0 V to 120 V, the electrical transport characteristics of the device continue to increase, and it drops significantly at the pulse amplitudes of 150 V. This shows that under the stimulation of 80 V electric pulses, the device is in good condition.

② Pulse frequency: As shown in **Supplementary Fig. 21**, by controlling the time interval between two electric pulses, we changed the period time (or frequency) of the electric pulses, and measured its influence on the electrical transport of the device. **Supplementary Fig. 21a** shows the electric pulses with voltage of 80 V, pulse width of 33 ms, and various frequencies of 20 Hz, 10 Hz, 5 Hz, 1 Hz, 0.5 Hz, and the corresponding currents. As can be seen, increasing the frequency (reducing the period time) of the electric pulses can effectively improve the increasing rate of the current. In addition, **Supplementary Fig. 21b** summarizes the recovery curves of the current labeled ‘1’ to ‘5’ in **Supplementary Fig. 21a**, indicating that improving the frequency (reducing the period time) of the electric pulse can enhance the initial current of the device after stimulations. We also plotted the required time of electric pulse stimulation on the device to tune the device current to reach 18.7 μA in **Supplementary Fig. 21c**. It can be observed that the modulation of 20 Hz electric pulse (period time: 50 ms) on the device for 0.23 s is equivalent to that of 0.5 Hz electric pulse (period time: 2 s) for 20.1 s. This means that a high frequency electric pulse with short stimulation duration

can make a same effect on the device as a low frequency electric pulse with long stimulation duration. Therefore, reducing the period time of the electric pulse can making the modulation of electric pulse on the device equivalent to that of a long pulse condition.

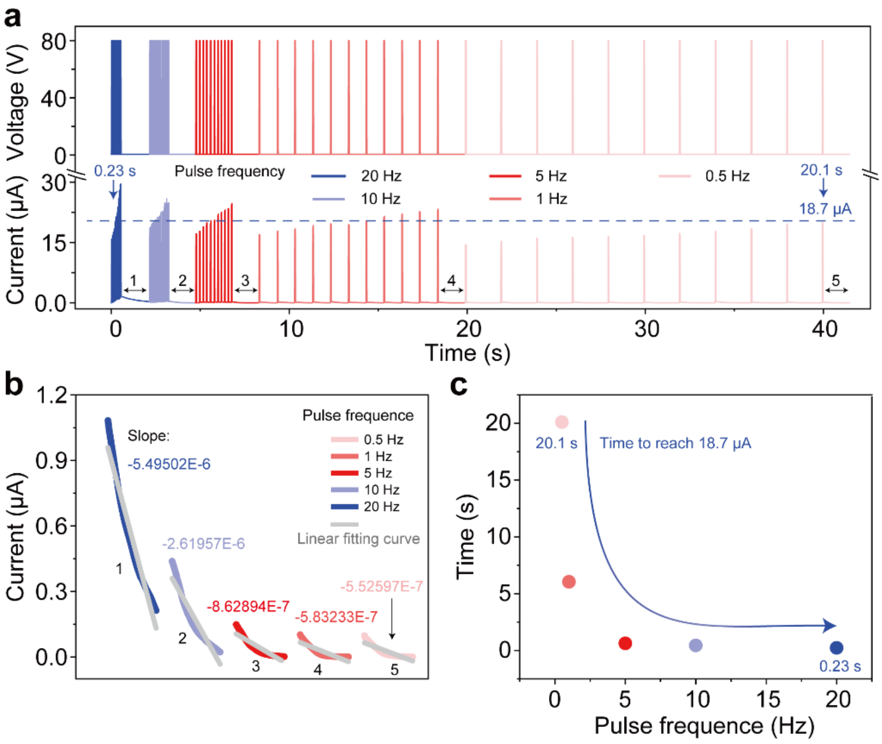

Supplementary Fig. 21 | Current response of the device under stimulations of electric pulse with different pulse frequency of 0.5 Hz, 1 Hz, 5 Hz, 10 Hz and 20 Hz. The pulse frequency of 0.5 Hz, 1 Hz, 5 Hz, 10 Hz and 20 Hz also present the period time of 20 s, 1 s, 0.2 s, 0.1 s, 0.05 s, respectively. **a**, Waveforms of the applied pulse voltage and measured response current over time. **b,** Recovery curves of the current labeled ‘1’ to ‘5’ in (**a**). The decreasing slopes of the current shown in (**b**) for each recovery curves have a unit of μm/s. **c**, Required time of electric pulse stimulation on the device to tune the response current to reach 18.7 μA. The frequency is regulated by changing the time interval between two electric pulses.

(2) Selection of pulsed electric fields between voltage source table and TENG

To clarify the mechanism of utilizing electric pulse stimulation to achieve tunable barrier of tunneling junction and thus regulate the piezotronic effect in the tunneling junction, the applied electric pulse needs to be stable and controllable. Although

TENG's output voltage can influence the barrier height, but it is not a good stimulation signal to modulate the barrier, especially it is impossible to use it to study the inner mechanism of the modulation. The TENG's output not only depends on the mechanical stimuli (amplitude, frequency, width) but also depends on the external resistance (the resistance of device or interface barrier in this work), leading to inconsistent peak outputs[30-35]. This instability hinders the precise investigations into the effect of steady pulse amplitude on oxygen vacancies and their impact on the interface barrier. Moreover, the transient nature and limited pulse width of output voltage confine the scope for controllable pulse width modulation. Coupled with a low adjustable frequency range, the TENG is hard to achieve high frequency electric pulse. These reasons make it temporarily difficult to carry out experiments with TENG. Of course, we believe that with the deepening of research, TENG will provide a highly programmable pulse voltage source. Utilizing an external pulse source to generate electric pulse offers enhanced stability, including amplitude consistency and temporal continuity. This approach facilitates the precise control over amplitude, frequency, pulse width, amplitude voltage duration, and pulse period duration, enabling transient and sustained electric pulse. This versatility is instrumental in examining the dynamics of electric pulse stimulation, including the effects of time, frequency, and amplitude, on the modulation of the interface barrier, which is critically important for us to find the typical modulation of piezotronics. And the finding of obvious modulation and the effective technical way for this modulation constitute the important progress.

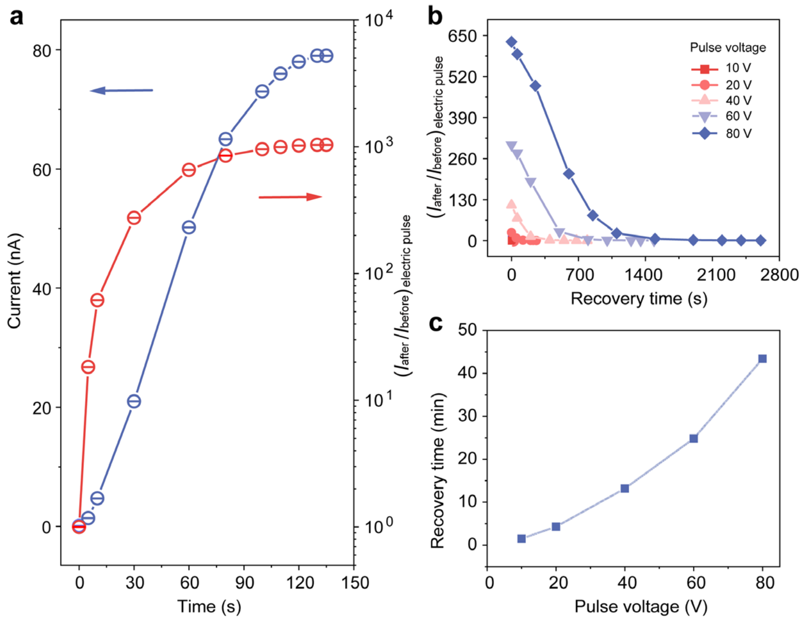

Supplementary Fig. 22 | Influence of electric pulse voltage on current characteristics. a, The relationship of the stimulation time of the electric pulse voltage (80 V) and the output current, as well as the current change ratio $(I_{after}/I_{before})_{electric\ pulse}$ caused by electric pulses, I_{after} represents the response current after pulse voltage stimulation, I_{before} represents the current without electric pulse stimulation. **b,** The change of current change ratio $(I_{after}/I_{before})_{electric\ pulse}$ as the function of the recovery time. **c,** Summary of the recovery time after different electric pulse voltage stimulation.

Supplementary Note 11 | In-situ measurement of tunable interfacial barrier height by AFM

We further performed in-situ measurement by atomic force microscope (AFM) to verify the tunable interface barrier height. Introducing programmed pulse voltages by the model of piezo-response force microscope (PFM model in AFM) and measuring the current by the model of conductive atomic force microscope (CAFM model in AFM), we can stimulate the nanowires firstly and perform subsequent *I*-*V* characterizations in situ. **Supplementary Fig. 23a** presents the optical image of the ZnO nanowire during in-situ measurement. **Supplementary Fig. 23b-e** shows the waveforms of periodic pulse voltages with amplitudes of 4 V, 6 V, 8 V, 10 V, and a frequency of 100 Hz. Following a pulse duration of 10 s, we carried out the *I*-*V* characteristics of the nanowire.

As shown in **Supplementary Fig. 23f**, a clear presence of tunable current was observed, with the increment of pulse voltage amplitudes. This indicates that electric pulse stimulation can effectively tune the interface barrier, and thus achieves tunable interface barrier height.

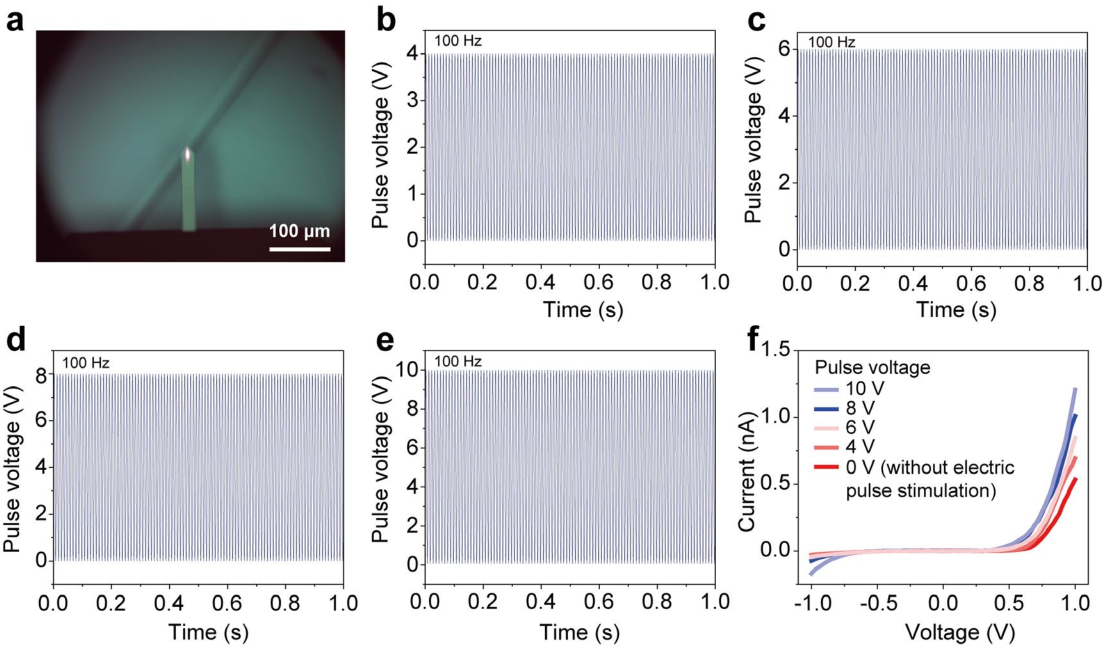

Supplementary Fig. 23 | In-situ measurement of tunable interfacial barrier height by AFM. a, Optical image of ZnO nanowire during in-situ measurement. **b-e**, Applying periodic pulse voltages with amplitudes of 4 V, 6 V, 8 V, 10 V, and a frequency of 100 Hz on the ZnO nanowire. **f**, *I-V* characteristics measured after various electric pulse voltages.

Supplementary Note 12 | Reason for using low sweeping voltage

In piezotronics, high voltage will lead to degraded piezotronic modification, so *I-V* characteristics of piezotronic devices are typically measured at low sweeping voltage. Also, the objective of performing *I-V* measurements is to evaluate the impact of piezotronic effect before and after electric pulse. The *I-V* curves measured at low voltage fulfill the research requirements. Moreover, high sweeping voltage may lead to damage to the interface barrier. Therefore, we only measured the *I-V* characteristics at low voltages. Additional experiments and explanation are as follows.

Supplementary Fig. 24 shows the comparison of strain-controlled *I-V*

characteristics at different sweeping voltages (±1 V, ±3 V, ±5 V, ±7 V and ±9 V). It shows an increased current under both strains of 0.00% and -0.10% as the sweeping voltage increases, indicating low interface barrier height under a high sweeping voltage. Because the interface barrier almost disappears under high sweeping voltage, the piezotronic effect is weakened. As shown in **Supplementary Fig. 24e**, the *I-V* curves for 0.00% and -0.10% almost overlap with each other when the sweeping voltage is ±9 V. **Supplementary Fig. 24f** summarized the current on-off ratio as a function of the sweeping voltage, indicating a degraded piezotronic effect at high sweeping voltage.

Supplementary Fig. 25 shows the *I-V* curves at ±1 V and ±5 V sweeping voltages after electric pulse (80 V and 10 Hz) with durations of 0 s, 30 s, 60 s and 120 s, respectively. Under ±1 V sweeping voltage, the current gradually rises with more time stimulation (**Supplementary Fig. 25a**). While, under ±5 V sweeping voltage, the current increases in the range of 0-60 s but suddenly decreases at 120 s (**Supplementary Fig. 25b**). This indicates that high sweeping voltage testing can potentially induce damage to the interface.

The above results suggest that higher sweeping voltages are not ideal for studying the tuning of electric pulses on piezotronics. So, we opted to examine the *I-V* characteristics at lower voltages in this work.

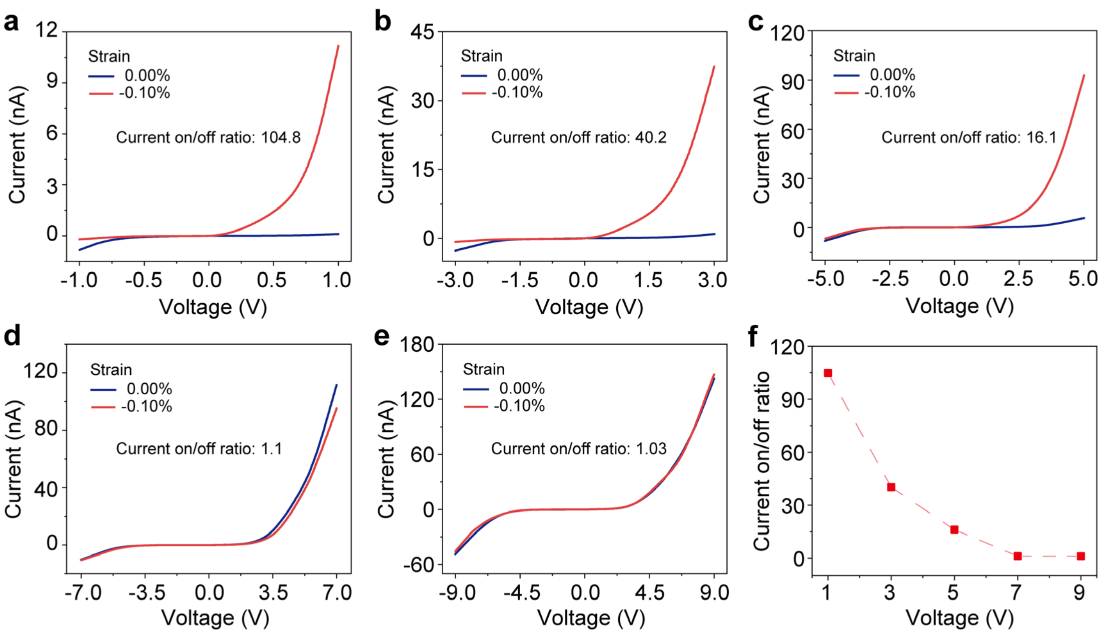

Supplementary Fig. 24 | Comparison of *I-V* characteristics at different sweep voltage of Ag/HfO_2/*n*-ZnO device. a-e, The *I-V* curves is measured at different sweep voltage of ±1 V (**a**), ±3 V (**b**), ±5 V (**c**), ±7 V (**d**) and ± 9 V (**e**), respectively, under the compressive strain of 0.00% and 0.10%. **f**, Relationship between current on/off ratio and sweep voltage.

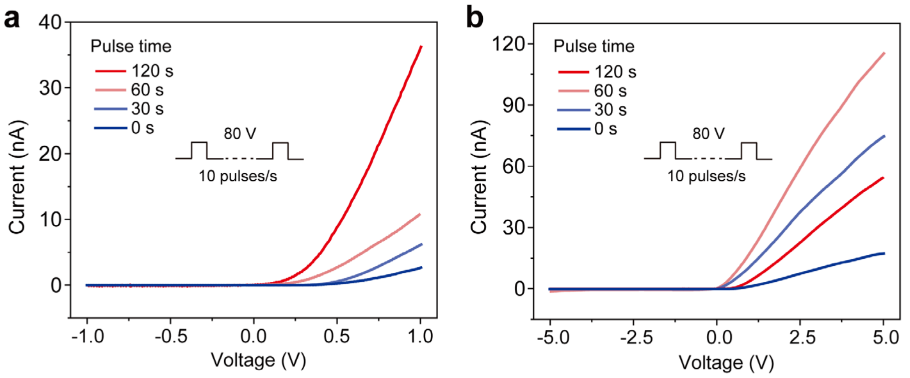

Supplementary Fig. 25 | *I-V* characteristics under electric pulse stimulations with different duration at sweep voltages of ±1 V and ±5 V. a, **b**, *I-V* curves under electric pulse stimulations for 0 s, 30 s, 60 s and 120 s measured at sweep voltages of ±1 V (**a**) and ±5 V (**b**), respectively. The electrical pulse used has a frequency of 10 Hz, and the amplitude is set at 80 V.

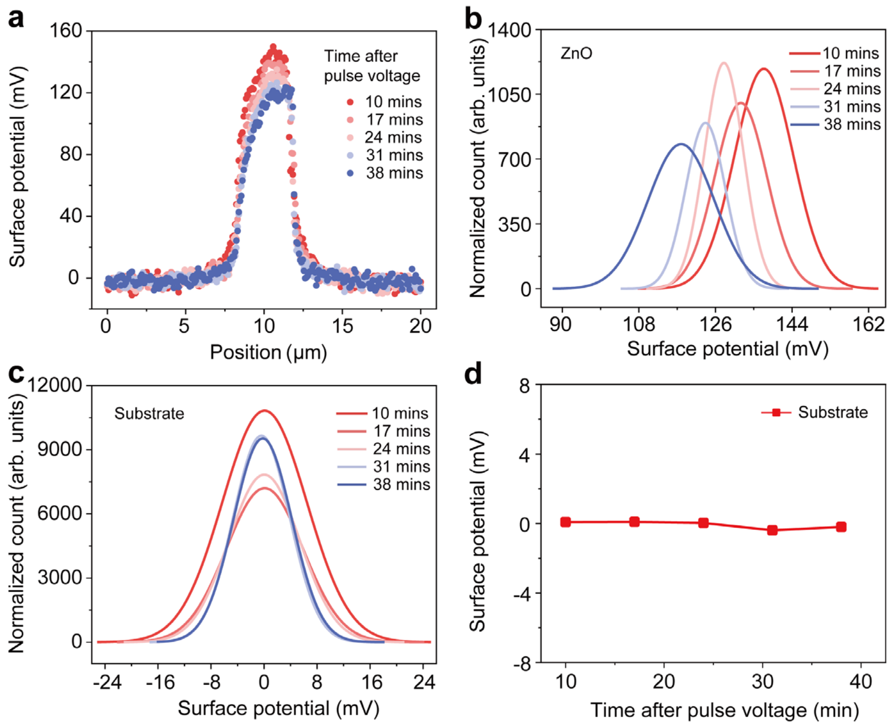

Supplementary Fig. 26 | Surface potential characterized by SKPM after electric pulse voltage stimulation. a, Surface potential measured at 10 mins, 17 mins, 24 mins,

31 mins and 38 mins after the voltage pulse stimulation, respectively. **b,** Statistical distribution of surface potential of ZnO. **c** and **d,** Statistical distribution of surface potential of the substrate in two forms.

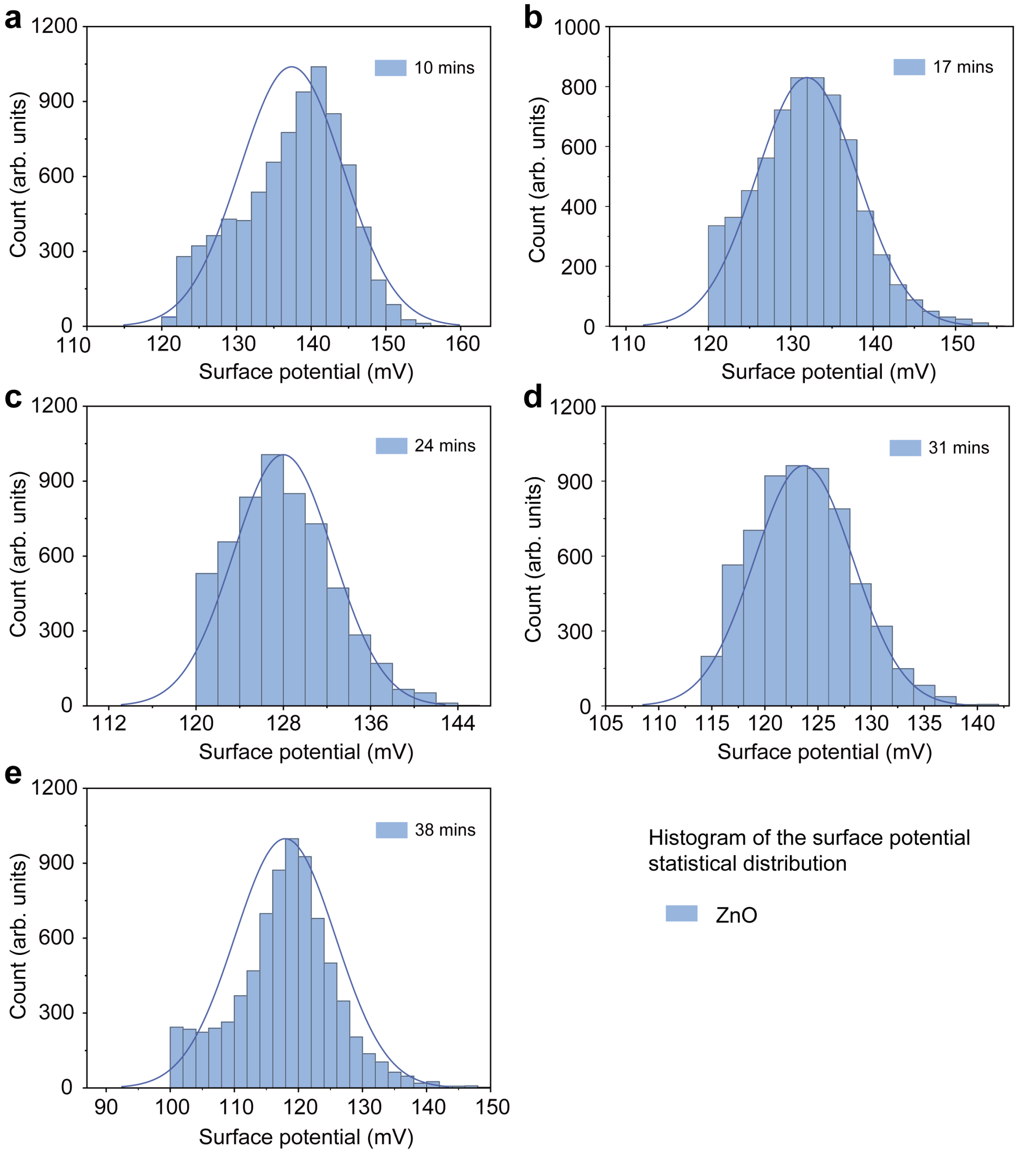

Supplementary Fig. 27 | Histogram of surface potential statistical distribution of ZnO. a-e, Surface potential measured at (**a**) 10 mins, (**b**) 17 mins, (**c**) 24 mins, (**d**) 31 mins and (**e**) 38 mins, respectively, after the electric pulse stimulation.

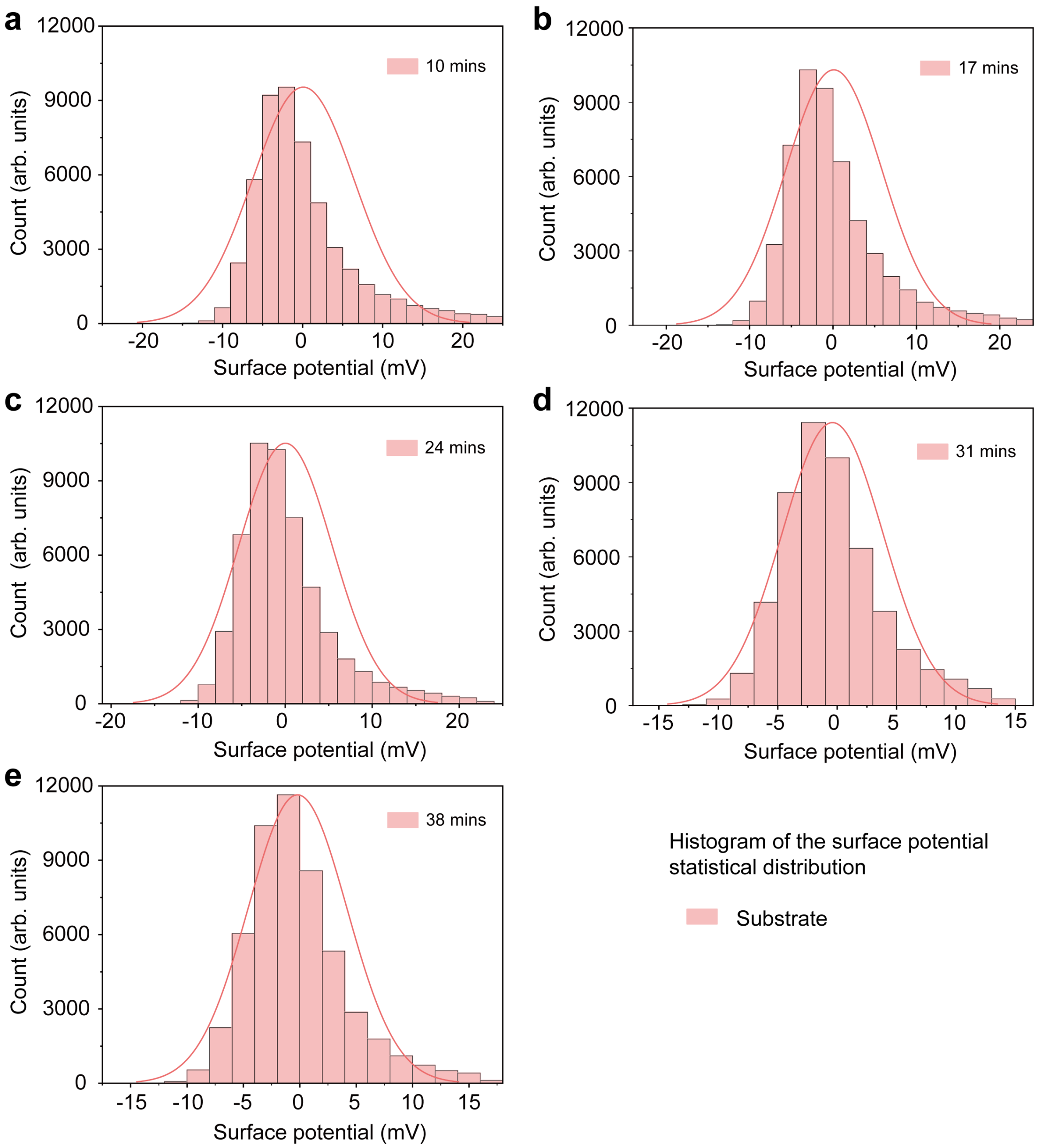

Supplementary Fig. 28 | Histogram of surface potential statistical distribution of the substrate. a-e, Surface potential measured at (**a**) 10 mins, (**b**) 17 mins, (**c**) 24 mins, (**d**) 31 mins and (**e**) 38 mins, respectively, after the electric pulse stimulation.

Supplementary Note 13 | Characterization of surface potential by SKPM

In this work, the effect of electric pulse voltage stimulation on the surface potential of ZnO nano/microwire was studied by atomic force microscopy. During the experiment, we did not test the surface potential of ZnO at the interface, but at a position slightly away from the interface, as shown in **Supplementary Fig. 29a.** For semiconductors, its work function W_s is similarly defined as the difference between the vacuum energy level E_0 and the Fermi energy level E_{fs} (**Supplementary Fig. 29b**), which can be expressed as:

$$W_s = E_0 - E_{fs} \tag{4}$$

Scanning kelvin probe microscope (SKPM) in atomic force microscope is a technology to detect the potential difference between the probe tip and the sample, which can measure the work function (surface potential) of the sample. The technique relies on an AC bias to produce an electric force on the cantilever, which is proportional to the potential difference between the tip and sample. During the nap pass, there is no mechanically induced drive (such as with a drive piezo in standard AC mode imaging or in Electric Force Microscopy). The only oscillation of the probe will be induced by the application of AC bias[36].

An AC bias applied between the tip and the sample produces an electrostatic force between the two. If they are modeled as a parallel plate capacitor, then the force between the two plates is proportional to the square of the applied voltage[36, 37]:

$$F = \frac{1}{2}\frac{\partial C}{\partial z}V^2 \tag{5}$$

The total potential difference between the probe and the sample is the sum of the applied AC bias (V_{ac}), the potential difference (V_{sp}), and any DC voltage (V_{DC}).

Then, we can get,

$$V^2 = \left(V_{DC} - V_{sp}\right)^2 + 2\left(V_{DC} - V_{sp}\right)V_{ac}sin(\omega t) + {V_{ac}}^2 sin(\omega t)^2 \tag{6}$$

Because

$$sin^2\theta = \frac{1}{2}(1 - \cos(2\theta)) \tag{7}$$

We have

$$V^2 = \left(V_{DC} - V_{sp}\right)^2 + 2\left(V_{DC} - V_{sp}\right)V_{ac}sin(\omega t) + \frac{1}{2}{V_{ac}}^2(1 - \cos(2\omega t)) \tag{8}$$

which shows the important point, that there are DC, $1\omega t$ and $2\omega t$ components to the signal. Putting this into equation (5) and a little rearranging gives us:

$$F = \frac{1}{2}\frac{\partial C}{\partial z}\left(\left[\left(V_{DC} - V_{sp}\right)^2 + \frac{V^2{}_{ac}}{2}\right] + 2\left[\left(V_{DC} - V_{sp}\right)V_{ac}sin(\omega t)\right] - \left[\frac{V_{ac}{}^2 cos(2\omega t)}{2}\right]\right) \quad (9)$$

Note the $2F$ component may have a significant force, but is typically not enhanced by the resonance of the cantilever, meaning that the cantilever does not respond to that frequency. The main point to SKPM is the middle part of this equation, which shows the minimized force (amplitude) if $V_{DC} - V_{sp} = 0$ or $V_{DC} = V_{sp}$. SKPM uses a feedback loop to adjust the DC bias on the lever to minimizing amplitude.

Because the electric pulses stimulation will regulate the barrier height (**Supplementary Fig. 29c**), that is, the work function (surface potential) of ZnO will change. In order to make the test data more accurate, we use the change of surface potential in a certain time to study the effect of electric pulse voltage on the device during the recovery process of the device after the electric pulses stimulation.

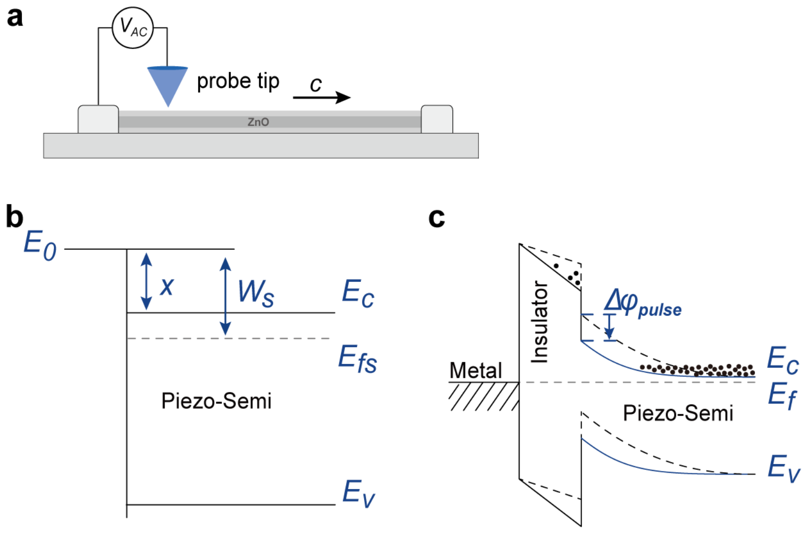

Supplementary Fig. 29 | Measurement of scanning kelvin probe microscope. a, Simplified test diagram of scanning kelvin probe microscope (SKPM). **b,** Energy band diagram of semiconductor. **c,** Regulation of electric pulse voltage on interface barrier.

Supplementary Note 14 | Calculation of the strain of the devices

For strain controlled piezotronic devices, the accurate strain calculation is helpful to

analyze the correct working mechanism. After repeated stretching and compression of the device, we find that PDMS has good toughness under all stress states, indicating the excellent stability of PDMS as a buffer material. Then, we analyzed the reason: Young's modulus of PET (3-3.5 GPa) is much higher than that of PDMS (360-870 KPa), so the mechanical properties of PET are not affected by PDMS. As shown in **Supplementary Fig. 30a**, during the experiment, we use PET as a flexible substrate, and use Ag electrodes to fix both ends of the nano/microwires. Finally, we use PDMS to encapsulate the entire device to stabilize the toughness of the nano/microwire and protect it from air. In this work, the regulation of mechanical strain on device performance was investigated, according to the regulating mechanism of piezoelectric charges on interface barrier (**Supplementary Fig. 30b**). Considering that the area and thickness of Ag electrode and the size of ZnO nano/microwires are much smaller than that of PET substrate, the strain of nano/microwire can be obtained by calculating the strain of substrate. As shown in **Supplementary Fig. 30c**, where mechanical strain occurs on the device, M represents the relative displacement of the two ends of the device, and the distance between the two ends of the device is represented by L. Assuming that the bending radius of the strain device is R, the formula of R can be expressed as follows[38]:

$$R = (L^2 + 4M^2)/8M \tag{10}$$

We assume that the length of the nano/microwire is l, its diameter is d, and the thickness of the PET substrate is h. Since R is much larger than h, and h is much larger than d, we can approximate the strain of the nano/microwires (ε) to that of the outer surface of the substrate. The expression formula of strain ε is obtained:

$$\varepsilon = h/2R = 4Mh/(L^2 + 4M^2) \tag{11}$$

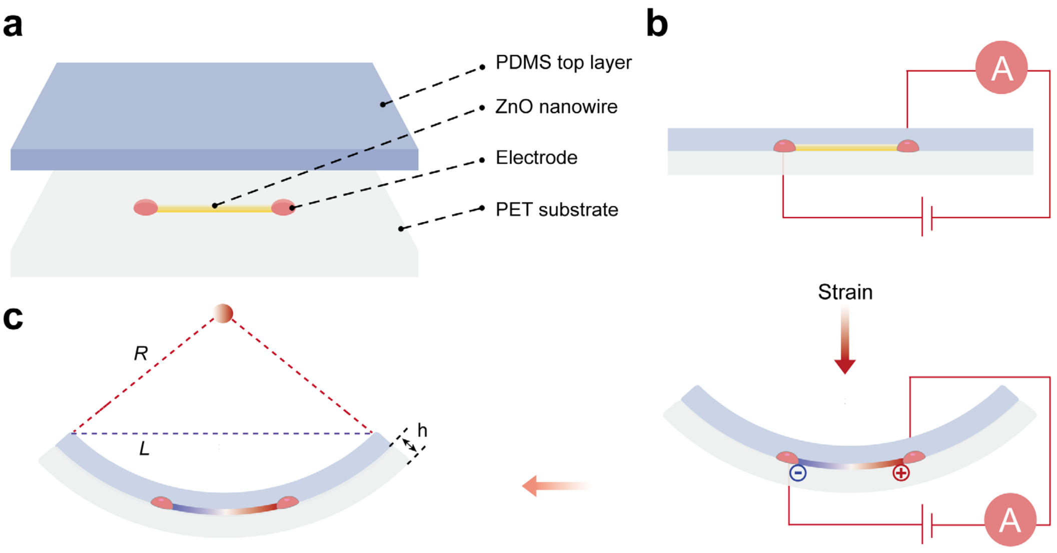

Supplementary Fig. 30 | Schematic diagrams of device structure and working process. a, Structure drawing of the device. **b,** Schematic of the measurement system to characterize the performance of the device. **c,** Schematic diagram of the device with a bending radius *R*.

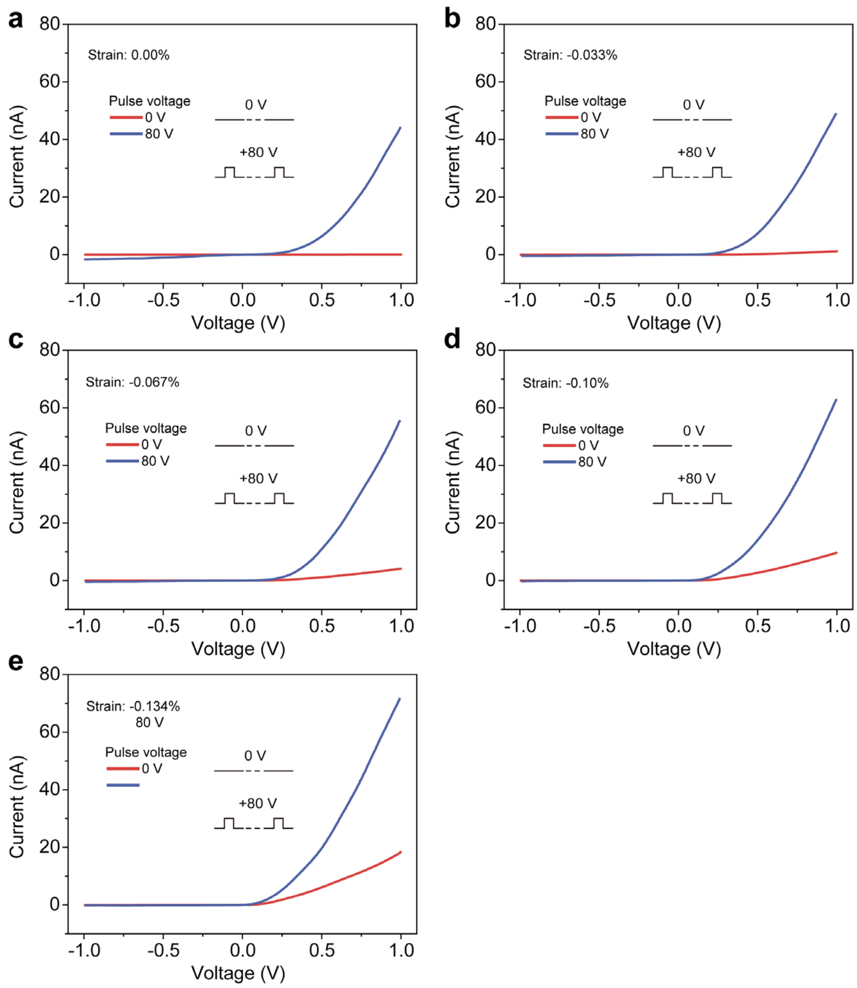

Supplementary Fig. 31 | Comparison of *I-V* characteristics at different strain with 0 V and 80 V electric pulse voltage stimulation. a-e, The *I-V* test is based on the strain of 0.00%, -0.033%, -0.067%, -0.10% and -0.134%, respectively.

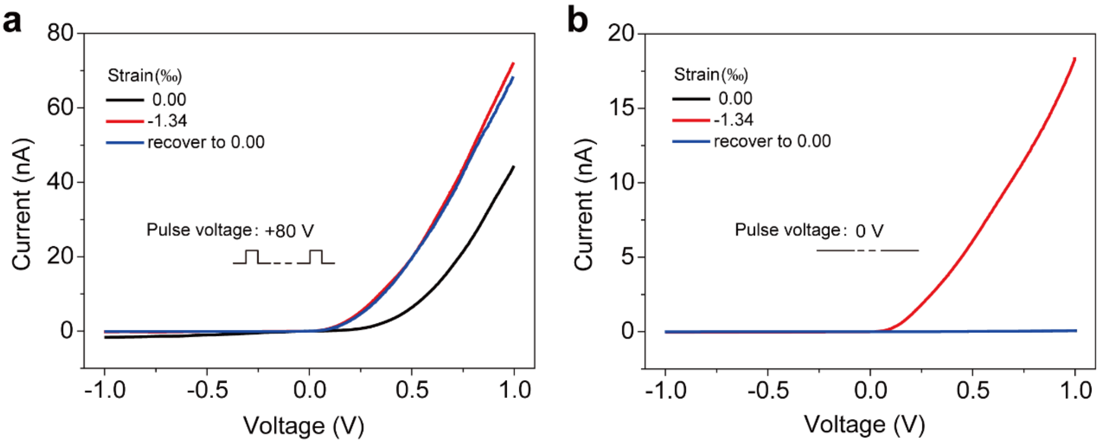

Supplementary Fig. 32 | *I-V* characteristics under the strain of 0.00%, -0.134% and no strain recovery state. a, b, Corresponding electric pulse voltage is 80 V (**a**) and 0 V (**b**), respectively.

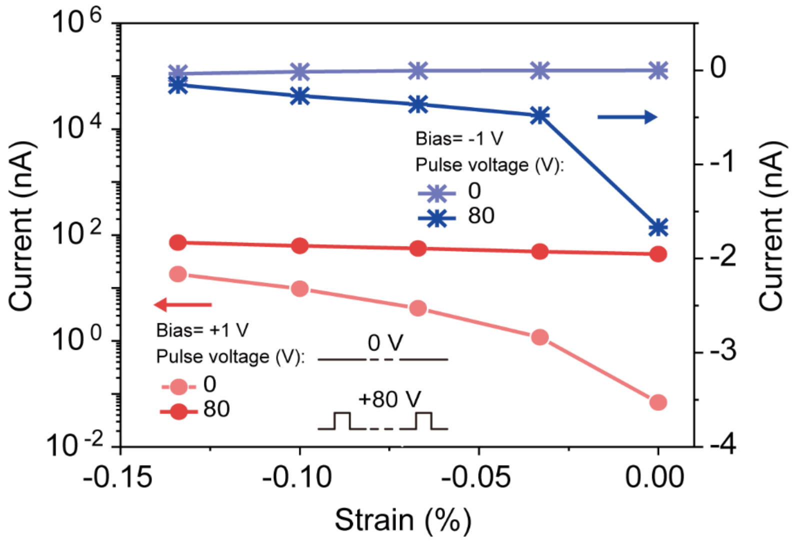

Supplementary Fig. 33 | Current as a function of the applied strain with the electric pulse stimulation.

Supplementary Note 15 | Definition of the current change ratio

Here, the current change ratio reflects the ability of electrical pulse voltage to modulate the device performance, which is defined as the ratio of the current I_{after} (corresponding to the response current after the stimulation of electric pulse) to the

current I_{before} (corresponding to the current before the stimulation of electric pulse), which can be written as

$$current\ change\ ratio = (I_{\text{after}}/I_{\text{before}})_{\text{electric pulse}} \quad (12)$$

Supplementary Note 16 | Calculation of the change of effective Schottky barrier height

The energy band structure of the heterogeneous interface formed III-V group semiconductor and metal can be changed by strain induction, and the Schottky barrier height can be tuned by the piezoelectric effect of the semiconductor material. Under certain conditions, this phenomenon can also be used to study the relationship between semiconductor carrier concentration and device performance. This is mainly because the piezoelectric charges cause the local Fermi level to shift and change the local band structure. For example, when the stress is applied to a piezoelectric semiconductor device, the resulting negative polarization charge makes the Schottky barrier rise and makes the barrier approach a high resistance state, resulting in a decrease in the electrical output characteristics of the device. The resulting positive piezoelectric charge reduces the Schottky barrier and makes the barrier close to the low resistance state, thus improving the electrical output characteristics of the device. The Schottky barrier plays an important role in the electrical transport characteristics of MSM devices. The electrical performance of the device is mainly determined by the reverse-biased Schottky junction, which also determines the *I-V* characteristics of the piezotronic device due to its high resistance and high partial voltage. According to the classic thermionic emission-diffusion theory (for $V \gg 3kT/q{\sim}77\ mV$), the current through the reverse-biased Schottky barrier can be obtained by the following formula[1]:

$$I = SA^{**}T^2 exp\left(-\frac{\varphi_S}{kT}\right) exp\left(\frac{\sqrt[4]{q^7 N_D (V + V_{bi} - kT/q)/(8\pi^2 \varepsilon_S^3)}}{kT}\right) \quad (13)$$

wherein, S is the area of Schottky barrier, A^{**} is the effective Richardson constant of ZnO nano/microwires, φ_S is the reverse biased Schottky barrier, q is the amount of electronic charge, and k is the Boltzmann constant. N_D is the donor impurity density, V_{bi} is the built-in potential in the junction barrier, and ε_S is the dielectric constant of ZnO.

The change of Schottky barrier height $(\Delta\varphi_{piezo})$ caused by piezoelectric charge will be got from the following equation[39, 40]:

$$ln[I_{strain}/I_{free}] \sim \Delta A^{**}/A^{**} - \Delta\varphi_{piezo}/kT \tag{14}$$

Under small deformation, we ignore ΔA^{**}, and assume that S, A^{**}, T, N_D are known. Considering that the measurement is carried out at room temperature, we can express the change of Schottky barrier height as follows:

$$\Delta\varphi_{piezo} = -kTln[I_{strain}/I_{free}] \tag{15}$$

wherein, I_{strain} and I_{free} represent the current under strain and strain-free conditions, respectively.

According to previous reports, the change of Schottky barrier height $(\Delta\varphi_{piezo})$ caused by strain is linearly related to the piezoelectric charge density ρ_{piezo}.

$$\Delta\varphi_{piezo} \approx -\frac{q\rho_{piezo}W_{piezo}^2}{2\varepsilon_S} \tag{16}$$

In this experiment, ZnO nano/microwires are strained along the *c*-axis, $\Delta\varphi_{piezo}$ can also be expressed as:

$$\Delta\varphi_{piezo} \approx -\frac{q\rho_{piezo}W_{piezo}^2}{2\varepsilon_S} = -\frac{qe_{33}\varepsilon_{33}\,W_{piezo}}{2\varepsilon_S} \tag{17}$$

Thus,

$$\Delta\ln(I) \propto \Delta\varphi_{piezo} \propto \rho_{piezo} \propto strain \tag{18}$$

Based on the derivation of the above formula, we can conclude that the change of Schottky barrier height can be controlled by strain induced piezoelectric polarization charge, and is linearly related to strain.

Supplementary Note 17 | Influence of the Ag/*n*-ZnO contact area of the side surface on the performance of the device

Since the metal electrodes contact the end face and side face of the ZnO nano/microwire at the same time, the calculated SBH change will be smaller than the actual value. Next, we will give relevant calculations to analyze and prove the influence of Ag/ZnO side contact area on the performance of the piezotronic device.

In **Supplementary Fig. 34a**, we assume that the radius of the end surface of the ZnO nano/microwire is r, and the length of the side contact area between ZnO nano/microwire and metal electrode is L'. Therefore, the area of the end surface (S_1) and side surface (S_2) can be expressed as,

$$\begin{cases} S_1 = \pi r^2 \\ S_2 = 2\pi r L' \end{cases} \tag{19}$$

Because the resistance of the Schottky barrier formed on the end face and side face is in parallel, the equivalent circuit can be drawn in **Supplementary Fig. 34b**, and the total current flowing through the device is obtained as follows:

$$I = I_1 + I_2 \tag{20}$$

where, I_1 and I_2 represent the currents flowing through the end surface and side surface, respectively.

Based on the Schottky theory[1], when the device is in strain-free state, we can obtain the current of the device by the following formula ($V > 0.026$ V),

$$\begin{cases} I_1 = S_1 A^{**} T^2 e^{-\frac{q\varphi_{B_{n0}}}{kT}} e^{\frac{qV}{kT}} \\ I_2 = S_2 A^{**} T^2 e^{-\frac{q\varphi_{B_{n0}}}{kT}} e^{\frac{qV}{kT}} \\ I = (S_1 + S_2) A^{**} T^2 e^{-\frac{q\varphi_{B_{n0}}}{kT}} e^{\frac{qV}{kT}} \end{cases} \tag{21}$$

wherein, A^{**} is the effective Richardson constant, $\varphi_{B_{n0}}$ is the Schottky barrier height under strain-free state, q is the electron charge, k is Boltzmann constant, T is the absolute temperature, and V is the applied voltage.

As a strain is applied to the ZnO nano/microwire, the positive and negative piezoelectric charges generated at the ends of the nano/microwire will regulate the height of the Schottky barrier, due to the piezoelectric effect. It is noteworthy that the influence of the piezoelectric charges on the Schottky barrier formed by the contact of the side surface of ZnO and the electrode is small. Therefore, the strain modulated current can be expressed as[41]:

$$\begin{cases} I_{1,strain} = S_1 A^{**} T^2 e^{-\frac{q(\varphi_{B_{n0}} + \Delta\varphi_{piezo})}{kT}} e^{\frac{qV}{kT}} \\ I_{2,strain} = S_2 A^{**} T^2 e^{-\frac{q\varphi_{B_{n0}}}{kT}} e^{\frac{qV}{kT}} \\ I_{strain} = I_{1,strain} + I_{2,strain} = \left(S_1 e^{-\frac{\Delta\varphi_{piezo}}{kT}} + S_2 \right) A^{**} T^2 e^{-\frac{q\varphi_{B_{n0}}}{kT}} e^{\frac{qV}{kT}} \end{cases} \tag{22}$$

where $\Delta\varphi_{piezo}$ represents the actual change of the Schottky barrier height of the ZnO end surface.

However, in the experiment, we did not consider the influence of the side surface, and mistakenly thought that the measured current was:

$$I'_{strain} = (S_1 + S_2)A^{**}T^2 e^{-\frac{q\left(\varphi_{Bn0}+\Delta\varphi'_{piezo}\right)}{kT}} e^{\frac{qV}{kT}} \tag{23}$$

in which $\Delta\varphi'_{piezo}$ is the effective change of the Schottky barrier height.

By setting I_{strain} equal to I'_{strain}, we can build the relationship between $\Delta\varphi_{piezo}$ (the actual value of the change of the Schottky barrier height) and $\Delta\varphi'_{piezo}$ (the calculated value of the change of the Schottky barrier height from experimental data without considering the influence of the side surface), and evaluate $\Delta\varphi_{piezo}$ by $\Delta\varphi'_{piezo}$.

$$\left(S_1 e^{-\frac{\Delta\varphi'_{piezo}}{kT}} + S_2\right) A^{**}T^2 e^{-\frac{q\varphi_{Bn0}}{kT}} e^{\frac{qV}{kT}} = (S_1 + S_2)A^{**}T^2 e^{-\frac{q(\varphi_{Bn0}+\Delta\varphi_{piezo})}{kT}} e^{\frac{qV}{kT}}$$

$$\Rightarrow\ S_1 e^{-\frac{q\Delta\varphi'_{piezo}}{kT}} + S_2 = (S_1 + S_2)e^{-\frac{q\,\Delta\varphi_{piezo}}{kT}}$$

$$\Rightarrow \frac{S_1}{S_2} e^{-\frac{q\Delta\varphi'_{piezo}}{kT}} + 1 = \left(\frac{S_1}{S_2} + 1\right) e^{-\frac{q\,\Delta\varphi_{piezo}}{kT}} \tag{24}$$

Due to

$$\frac{S_1}{S_2} = \frac{\pi r^2}{2\pi r L'} = \frac{r}{2L'} \approx \frac{4\ \mu\text{m}}{2 \times 100\ \mu\text{m}} = 0.02 \tag{25}$$

we can obtain the following relationship,

$$e^{-\frac{q\Delta\varphi_{piezo}}{kT}} \approx \left(0.02 \times e^{-\frac{q\Delta\varphi'_{piezo}}{kT}} + 1\right)/1.02 \tag{26}$$

For the generated positive piezoelectric polarization charge, $\Delta\varphi'_{piezo} < 0$.

Through analysis, we can estimate the change in the height of the Schottky barrier on the end surface by $\Delta\varphi'_{piezo}$.

For example, if we obtain the $q\Delta\varphi'_{piezo} = -kT = -26$ meV, the actual change in the height of the Schottky barrier $q\Delta\varphi_{piezo} \approx -133.8$ meV. This value of approximately 100 meV is consistent with previous studies in magnitude[42, 43].

In other words, the existence of side contact between Ag and ZnO nano/microwire reduces the performance of the device, leading to the underestimate of the actual change

of SBH. Therefore, in our devices, the change in carrier transport characteristics caused by piezotronic interface effects should be stronger in our devices.

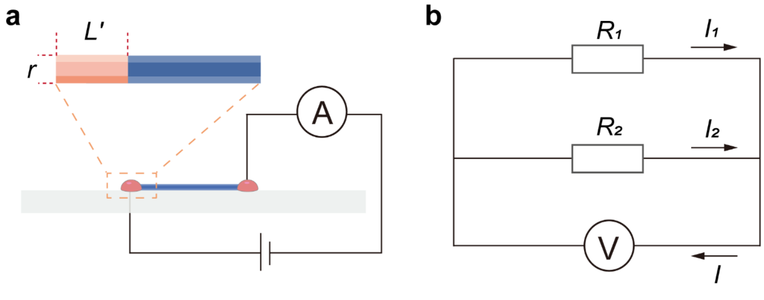

Supplementary Fig. 34 | Ag/*n*-ZnO contacts at end/side surfaces and the equivalent circuits. a, Side and end surfaces of ZnO nano/microwire in contact with metal electrodes. **b,** Equivalent circuit formed by the end surface contact resistance R_1 and the side surface contact resistance R_2.

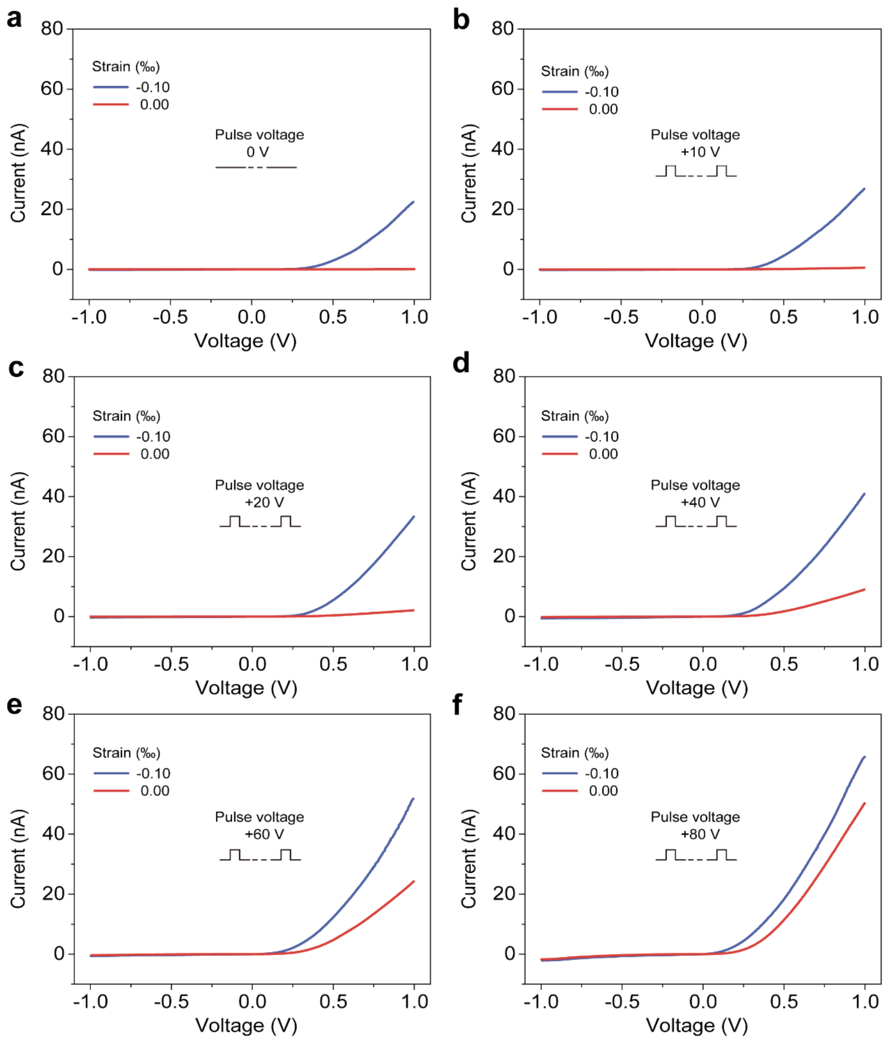

Supplementary Fig. 35 | Comparison of *I-V* characteristics under strain 0.00%

and -0.1% with electric pulse stimulation. a-f, The *I-V* test is based on the pulse voltage stimulation with different amplitudes of 0 V (**a**), 10 V (**b**), 20 V (**c**), 40 V (**d**), 60 V (**e**), and 80 V (**f**), respectively.

Supplementary Note 18 | The effect of post-annealing of as-prepared ZnO nanowires on the electric pulse-tuned piezotronic effect

We performed annealing on the ZnO nanowires in air at various temperatures: 200 °C, 300 °C, 400 °C, and 450 °C. And then, the *I-V* properties of devices with untreated and annealed ZnO were analyzed. **Supplementary Fig. 36a-e** show *I-V* curves for the devices under 0.00% and 0.10% compressive strain, revealing the existence of piezotronic effect at various annealing temperatures. At 0.10% compressive strain, higher current responses under positive bias and reduced responses under negative bias were observed. **Supplementary Fig. 36f** gives the changes in *I-V* curves, with peak current at 400 °C, influenced by oxygen vacancies on the Schottky barrier height. **Supplementary Fig. 36g** summarizes the current at ±1 V bias for 0.00% and -0.10% strain. The current increases with annealing temperature, indicating that annealing increases the carrier concentration and oxygen vacancy Vo^{2+} density. In addition, the current on/off ratio in **Supplementary Fig. 36h** decreases with temperature increasing from 0 °C to 400 °C. Modulation of Schottky barrier height in **Supplementary Fig. 36i** is consistent with the current on/off ratio trend. Results suggest that the impact of piezoelectric polarization on barrier height diminishes at 400 °C due to increased oxygen vacancy density and stronger carrier shielding. However, at 450 °C, a decrease in positively charged oxygen vacancies (Vo^{2+}, which will be transited into Vo^{0} above 400 °C) weakens this effect, resulting in higher current on/off ratios and Schottky barrier heights.

As illustrated in **Supplementary Fig. 37a**, we exposed the devices to electric pulse stimulation with an 80 V amplitude and a 10 Hz frequency. **Supplementary Fig. 37b-f** show the *I-V* characteristics of these devices both before and after a 60 s electric pulse stimulation. The measured *I-V* curves indicate that the electric pulse can significantly increase the devices' current. **Supplementary Fig. 37g** summarizes the relationship between the current value at +1 V and the annealing temperature. The increased current

before electric pulse stimulation indicates that the density of vacancy in *n*-ZnO nanowires increases with the increase of annealing temperature. Meanwhile, **Supplementary Fig. 37h** plots the current ratio before and after electric pulse as a function of the annealing temperature. Notably, this curve peaks at 400 °C, designating it as a critical inflection point, beyond which (at 450 °C) the current ratio significantly diminishes. The decreasing trend of current ratio indicates that with the increase of annealing temperature, the regulation ability of electric pulse tends to be weaker. This phenomenon can be easily understood as follows. As the annealing temperature increases, the density of vacancy as well as the free carrier concentration both increases. However, the increase of carrier concentration will electrostatically shield the vacancies and weaken the regulation of vacancy on the interface barrier. This phenomenon is similar to the weakened piezotronic effect or the weakened flexoelectronic effect occurred in high doping semiconductors. **Supplementary Fig. 37i** summaries the recovery time after electric pulse as a function of the annealing temperature. As can be found that the recovery time tends to increase with the increase of annealing temperature and reaches its peak value at 400 °C. This tell us that with the increase of vacancy density, the gradient of vacancy concentration after electric pulse stimulation is relatively small, and it will take more time to recover to its original state.

In summary, with the increase of post-annealing temperature, the vacancy Vo^{2+} concentration increases as a whole, which makes the device current increases (under a same bias) but weakens the interface engineering by the piezoelectric polarization and the electric pulse. These results from high vacancy Vo^{2+} concentration by post-annealing are very similar to the weakening of piezotronic effect caused by doping in the past.

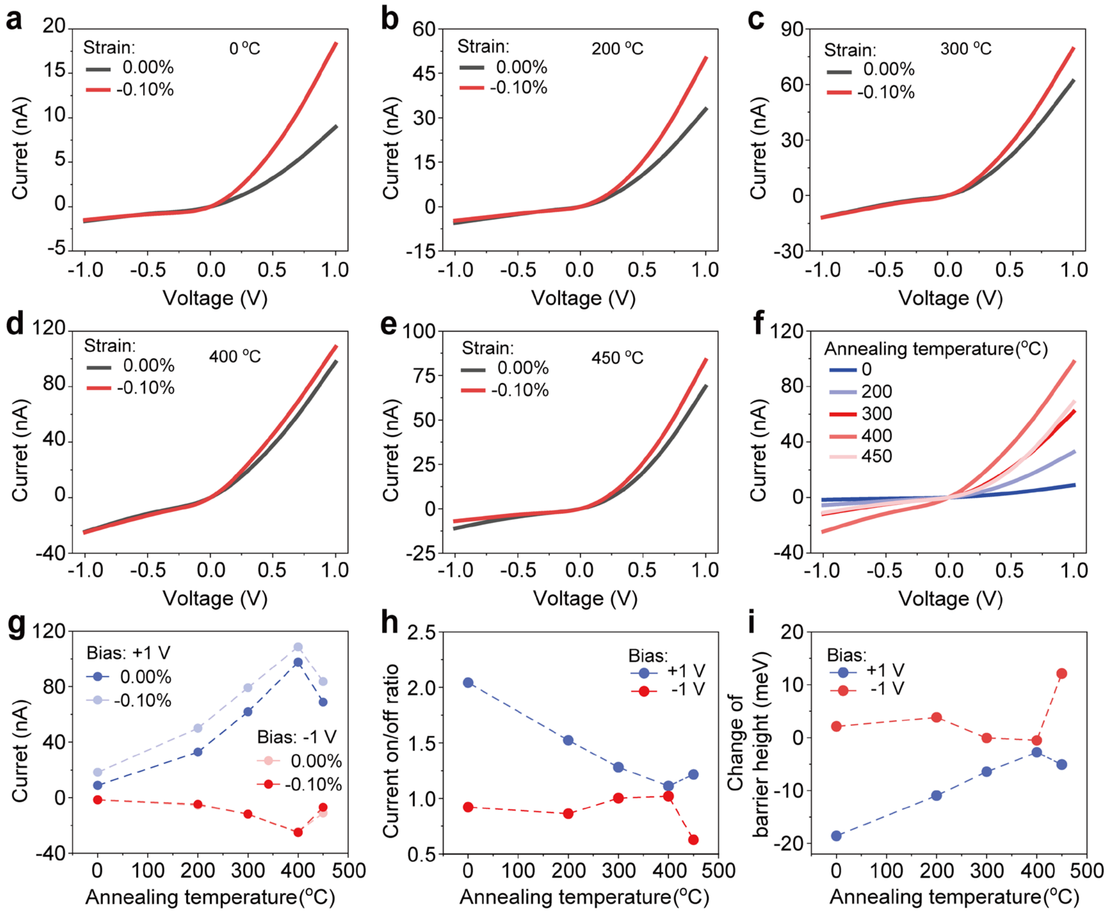

Supplementary Fig. 36 | Characterization of the influence of annealing temperature on piezotronic properties. a-e, *I-V* curves for Schottky barrier devices incorporating untreated and annealed ZnO nano/microwires, evaluated under two distinct compressive strain conditions: 0.00% and 0.10%. The annealing temperatures are 200 °C, 300 °C, 400 °C, and 450 °C, respectively. **f,** *I-V* characteristic trends across varying annealing temperatures. **g,** Current values under ±1 V bias for the 0.00% and 0.10% compressive strain conditions. **h**, **i**, Current on/off ratio (**h**) and change of Schottky barrier height (**i**) as functions of different annealing temperatures under ±1 V bias.

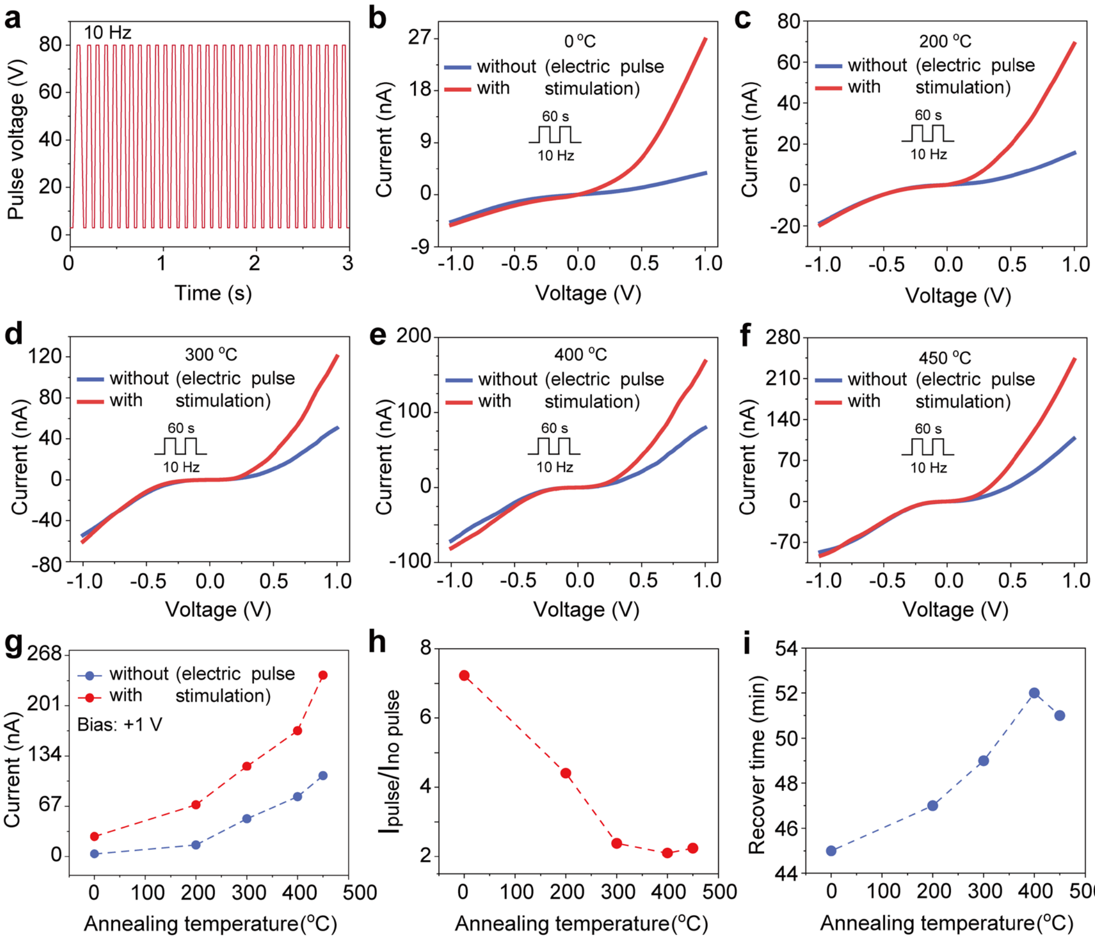

Supplementary Fig. 37 | Carrier transport characteristics before and after electrical stimulation after annealing. a, Electric pulse characterized by an 80 V amplitude and a 10 Hz frequency. **b-f**, *I-V* characteristics before and after electrical stimulation under different heating temperature. **g**, The relationship between the current value at a 1 V positive bias and the annealing temperature. **h**, The variations in current ratio before and after electrical stimulation as a function of annealing temperature. **i**, The recovery time of the device's current after electrical stimulation

Supplementary Note 19 | Theory of piezotronic effect on the tunneling junction

Based on the theoretical model of MIS junction tuned by piezoelectric polarization (**Supplementary Fig. 38**). When applying voltage to the MIS junction, there will be a partial voltage drop on the insulator, which can be written as:

$$\Delta = \frac{E_g}{q} + \chi_S - \phi_m - \phi_p - \psi_S + V \tag{27}$$

where E_g, χ_S and ϕ_m represents the semiconductor band gap, the affinity of the

semiconductor and the metal work function, respectively. ϕ_p represents the potential difference between the majority-carrier Fermi level and the valence band, ψ_S represents the potential across the semiconductor, and V is the applied voltage.

To simplify the analysis, we chose to ignore the surface conditions, work function differences, and other interferences of the MIS junction. In this process, we ground the semiconductor side and apply a positive voltage to the metal side. Next, assuming that the semiconductor operates in a thermal equilibrium state, direct tunneling is the main tunneling mechanism for carriers at the MIS interface. Therefore, the difference between the quasi-Fermi energy levels of electrons and holes can be ignored at this time. Thus,

$$\Delta = -\psi_S + V \tag{28}$$

According to the Gauss's law,

$$\Delta = E_i d_i = d_i \frac{Q_M}{\varepsilon_i} \tag{29}$$

here E_i, Q_M and d_i represents the electric field inside the insulator, the charge on the metal and the insulator thickness, respectively. ε_i represents the permittivity of the insulator.

To achieve the neutrality of the charge,

$$Q_M + Q_S + Q_{piezo} = 0 \tag{30}$$

wherein, Q_S represents the charge generated on the semiconductor surface due to ionization, and Q_{piezo} represents the piezoelectric polarization charge. Thus, Δ can be expressed as：

$$\Delta = -d_i \frac{\left(Q_S + Q_{piezo}\right)}{\varepsilon_i} \tag{31}$$

$$\mathrm{Q}_S = -qN_A W_{DP} \tag{32}$$

$$\mathrm{Q}_{piezo} = q\rho_{piezo} W_{piezo} \tag{33}$$

Based on the depletion approximation theory and the assumption of complete ionization in the depletion region, we can calculate the field and potential distribution in the MIS junction. Through the method of solving the one-dimensional Poisson's equation, we can have the potential $\psi_S(x)$ inside the semiconductor as a function of distance as:

$$\frac{d^2\psi_S(x)}{dx^2} = -\frac{dE}{dx} = -\frac{\rho(x)}{\varepsilon} = -\frac{q\left[p(x) - n(x) - N_A(x) + \rho_{piezo}(x)\right]}{\varepsilon} \tag{34}$$

where $\rho(x), N_A(x)$ *and* $\rho_{piezo}(x)$ represents the charge density, the density of the acceptor and the density of the piezoelectric charges, respectively. After integrating the Poisson equation, the electric field distribution inside the semiconductor can be obtained.

$$E(x) = -\frac{qN_A\left(x - W_{Dp}\right)}{\varepsilon_S} + \frac{q\rho_{piezo}\left(x - W_{piezo}\right)}{\varepsilon_S}\left(0 \le x \le W_{piezo}\right) \tag{35}$$

Then,

$$E(x) = -\frac{qN_A\left(x - W_{Dp}\right)}{\varepsilon_S}\left(W_{piezo} \le x \le W_{Dp}\right) \tag{36}$$

According to the above and through setting $\psi_S(N_A) = 0$, the potential distribution across the MIS junction can be given by

$$\psi(x) = \frac{qN_A\left(x - W_{Dp}\right)^2}{2\varepsilon_S} - \frac{q\rho_{piezo}\left(x - W_{piezo}\right)^2}{2\varepsilon_S}\left(0 \le x \le W_{piezo}\right) \tag{37}$$

$$\psi(x) = \frac{qN_A\left(x - W_{Dp}\right)^2}{2\varepsilon_S}\left(W_{piezo} \le x \le W_{Dp}\right) \tag{38}$$

Thus, the potential across the semiconductor changed by the band bending of the semiconductor valence band can be expressed as follows:

$$\psi_S = \psi(0) = \frac{q}{2\varepsilon_S}\left(N_A W_{Dp}^2 - \rho_{piezo} W_{piezo}^2\right) \tag{39}$$

where ε_S, N_A and ρ_{piezo} is the permittivity of the semiconductor, the acceptor concentration and the density of the piezoelectric polarized charges, respectively. W_{piezo} represents the width of the piezoelectric charges distribution region and W_{Dp} represents the depletion layer width on the semiconductor side.

According to the depletion assumption of the analytical model, it is known that most of the carriers have been removed in the depletion region. Then, the piezoelectric charges cannot be screened by free carriers. For piezoelectric regulated MIS structure, piezoelectric polarization can change the energy band and the built-in potential in the tunneling junction.

In the simplified analytical model, due to the neglect of carrier redistribution, according to a typical tunneling current model, hole and electron tunneling currents can

be expressed as:

$$J_{nt} = A_n^* T^2 \exp(-\alpha_{Tn}\varphi_{Tn}^{1/2} d_i) \left[\exp\left(-\frac{E_{c0} - E_{fm}}{kT}\right) - \exp\left(-\frac{E_{c0} - E_{fs}}{kT}\right)\right] =$$

$$A_n^* T^2 \exp(-\alpha_{Tn}\varphi_{Tn}^{1/2} d_i) \exp\left(-\frac{E_g}{kT}\right) \exp\left(\frac{q\phi_p + q\psi_S}{kT}\right)\left[\exp\left(\frac{qV}{kT}\right) - 1\right] \quad (40)$$

$$J_{pt} = A_p^* T^2 \exp(-\alpha_{Tp}\varphi_{Tp}^{1/2} d_i) \left[\exp\left(-\frac{E_{fs} - E_{v0}}{kT}\right) - \exp\left(-\frac{E_{fm} - E_{c0}}{kT}\right)\right] =$$

$$A_p^* T^2 \exp(-\alpha_{Tp}\varphi_{Tp}^{1/2} d_i) \exp\left(-\frac{q\phi_p + q\psi_S}{kT}\right)\left[1 - \exp\left(-\frac{qV}{kT}\right)\right] \quad (41)$$

where A_n^* and A_p^* are effective Richardson constants for electrons and holes, α_{Tn} and α_{Tn} equal to $2\sqrt{2qm_n^*}/\hbar$ and $2\sqrt{2qm_p^*}/\hbar$ (m_n^* and m_p^* are the effective mass of electrons and holes, $\hbar$ is reduced Planck constant), respectively. φ_{Tn} and φ_{Tp} are the effective barrier heights for electrons and holes tunneling into metal. The total current density can be given by

$$J_t = J_{pt} + J_{nt} \quad (42)$$

The above shows that the piezoelectric charges generated by force induction under mechanical stimulation can regulate the interface energy band and tunneling current J_t by changing the surface potential ψ_S of the semiconductor.

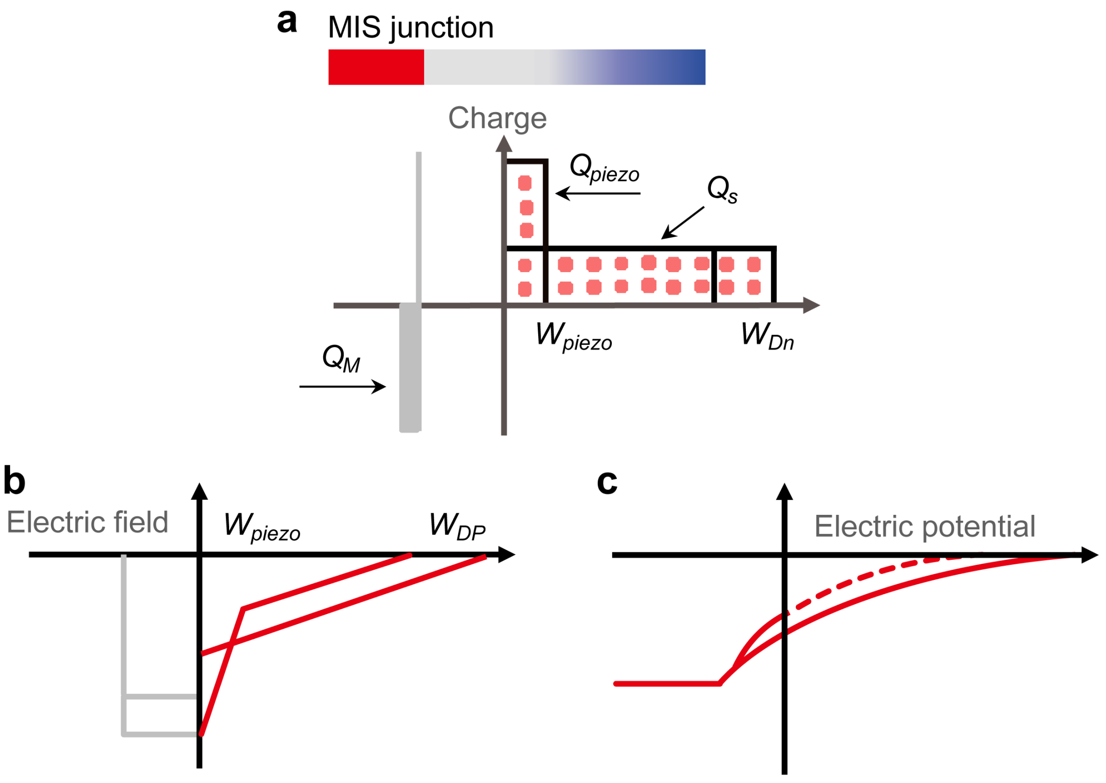

Supplementary Fig. 38 | Ideal MIS junction tuned by piezoelectric charges. a, b and **c** represent the charge distribution, electric field and the potential distribution, respectively.

Supplementary Note 20 | Regulation mechanisms of interface traps/defects on electrical transport under strain

By studying the possible influence of interface traps/defects on the electrical transport characteristics of devices, we can clearly distinguish the influence of piezotronic effect and interface traps, and finally determine the dominant regulatory mechanism in this work.

We will analyze the modulation of strain induced electrical transport by interface traps/defects. The interface traps have an important influence on the electrical transport properties of carriers at the interface. As strain is introduced, the interface trap states such as the concentration and distribution will also be changed, thus changing the effective resistance and electrical transport of the interface. The schematic diagram in **Supplementary Fig. 39** clearly shows how the interface trap affects the electrical transport of the device under strain in different conditions.

In **Supplementary Fig. 39a**, we assume that tensile strain increases the interfacial current and compressive strain decreases the interfacial current. When the device is stretched, the contact interface at both ends of the device will also be subject to tensile strain. Therefore, as shown in **Supplementary Fig. 39a**, no matter whether the voltage applied to the device is forward biased or reverse biased, the current will increase. It should be noted here that even if the contact states at both ends of the device are different, the forward and reverse currents of the device will still increase, but the amplitude of the current change may be different. Based on the above, the forward current and reverse current of the device will decrease under compression deformation. It can be clearly seen from the *I-V* curves in **Supplementary Fig. 39a** that the simultaneous increase or decrease of forward current and reverse current caused by strain (tensile or compress) is a symmetrical regulation.

In **Supplementary Fig. 39b**, we assume that the tensile strain-regulated interface current in the device decreases and the compressive strain-regulated interface current

increases. In this case, tensile strain decreases both forward and reverse currents, while compressive strain increases both. Thus, in the strain-regulated *I-V* curves shown in **Supplementary Fig. 39b**, the regulation of strain on interface traps and electrical transport of the device is still symmetric regulation.

In **Supplementary Fig. 39c**, we assume that both tensile and compressive strains increase interface carrier transport. In this case, the current of the device will increase no matter upon tensile strain or compressive strain. The corresponding *I-V* curves under strain regulation in **Supplementary Fig. 39c** also indicate a symmetric regulation.

In **Supplementary Fig. 39d**, we assume that both tensile and compressive strains reduce interfacial carrier transport. Similar to the case in **Supplementary Fig. 39c**, the forward and reverse currents decrease regardless of whether the device is under tensile or compressive strain. As shown in **Supplementary Fig. 39d**, the *I-V* curve is also symmetrically regulated by mechanical strain.

Through the above discussion and analysis, we can see that the strain induced regulation of electrical transport by interface trap is a symmetrical effect.

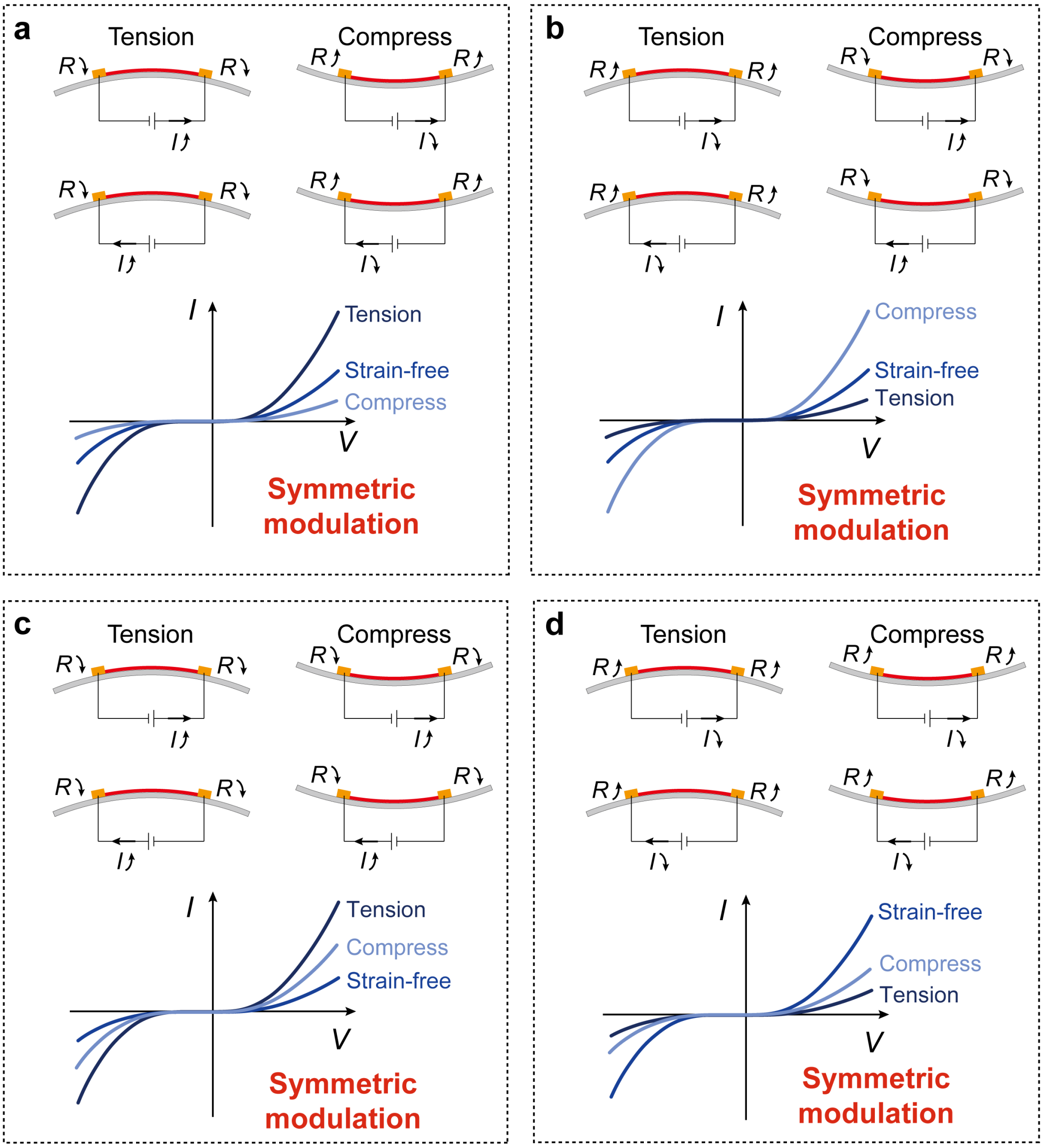

Supplementary Fig. 39 | Symmetric modulation of electrical transport by interface traps/defects under strain. a-d, The control mechanisms of strain on *I-V* characteristics under four different situations.

Supplementary Note 21 | Regulation mechanisms of piezoelectric charges on electrical transport under strain

Piezotronic effect is an interface effect, which regulates the electric transport of carriers at the interface through piezoelectric charge[39]. Next, we will analyze the strain-induced asymmetric modulation of electrical transport by piezoelectric charges.

As schematically illustrated in the **Supplementary Fig. 40**, the strain induced

interface piezotronic effect asymmetrically modulates local contacts at different terminals of the device. Under the condition of no strain, the tunneling junction is formed at two terminals of the device with local contacts (**Supplementary Fig. 40a**). The accidental error/uncertainty of device manufacturing makes the tunneling junctions (including barrier height and width) at both ends be slightly different. We assume that the piezoelectric *c*-axis of ZnO nano/microwire points to the left. When tensile strain occurs on the device (**Supplementary Fig. 40b**), positive piezoelectric polarization charges are generated on the left contact interface of the ZnO nano/microwire, which reduces the barrier height and width of the tunneling junction. While the negative piezoelectric polarization charges are generated at the right contact interface, resulting in the increased barrier height and width of the tunneling junction. When forward bias is applied (**Supplementary Fig. 40b-1**), the left terminal is in reverse bias state, and the electrical transport characteristics of the device are mainly determined by the left tunneling junction. As the barrier height and width of the left contact of the device with tunneling junctions are reduced, the current flowing through the device will increase, which is larger than that of the device without strain. Similarly, when the device is reversely biased (**Supplementary Fig. 40b-2**), the potential of right electrode is lower than that of left electrode in this case. Under a same bias voltage, the (reverse) current of the device will be smaller than that without strain. Therefore, compared to the case without strain, the forward current of the device with tensile strain will increase; while the reverse current will decrease. According to the above, as shown in **Supplementary Fig. 40c**, we can also analyze the modulation of the current by compressive strain based on the same principle. We can see that the compressive strain will cause the forward current to decrease (**Supplementary Fig. 40c-1**) and the reverse current to increase (**Supplementary Fig. 40c-2**). It can be seen that the interface modulation caused by strain induced piezoelectric charges (piezotronic effect) is asymmetric in the schematic illustrations of the *I-V* curves of the device under no strain (black), tensile strain (red) and compressive strain (blue) (**Supplementary Fig. 40d)**.

The piezotronic effect modulation is caused by the polarization of non-mobile ions in the piezoelectric crystal[7, 44], which is different from the strain-induced modulation by

interface defects/traps (**Supplementary Fig. 40**). After comparison, it can be seen that the strain induced modulation of electrical transport by interface defects/traps is a symmetrical effect due to the symmetrical changes at both ends of the device under strain: while piezotronic effect is an interface effect, which adjusts the local contact of different ends of devices asymmetrically by the polarity of piezoelectric potential.

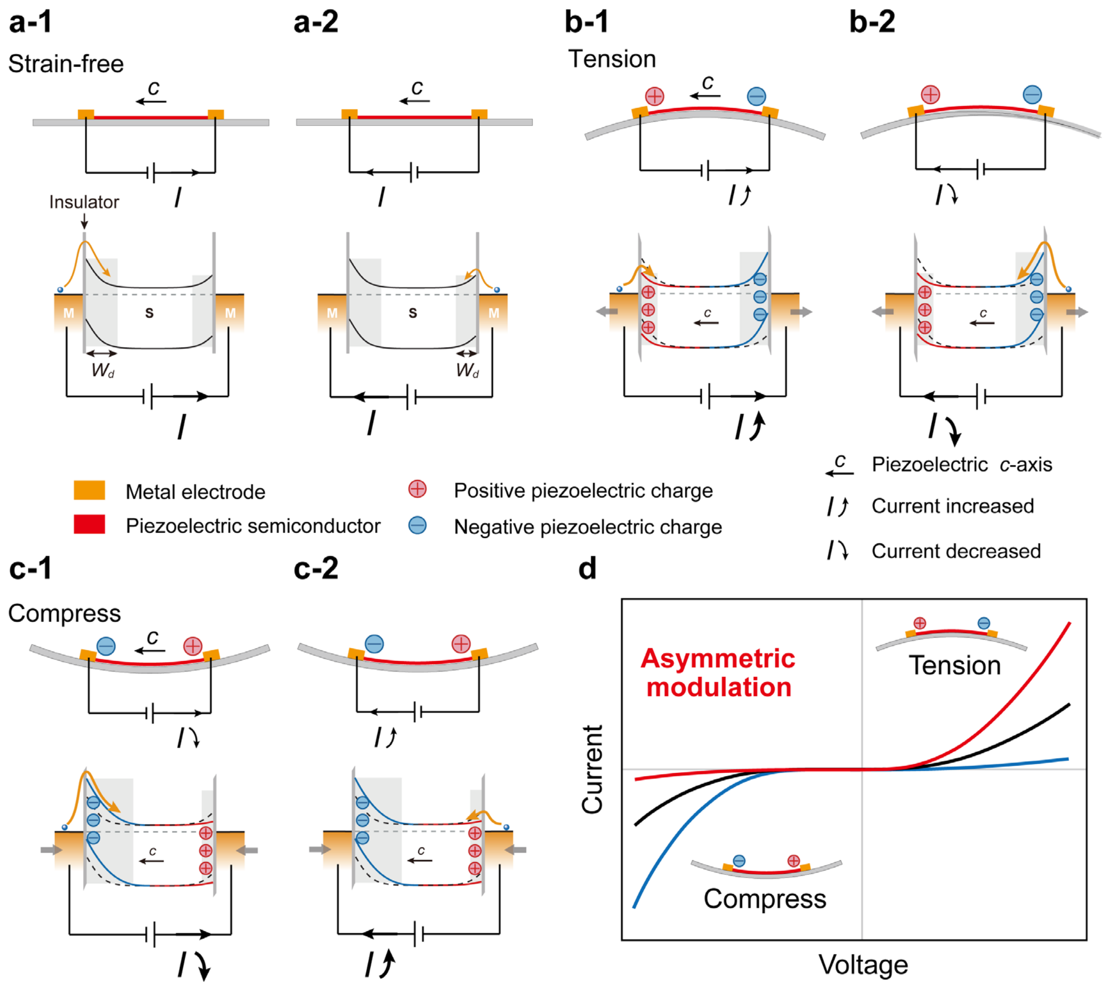

Supplementary Fig. 40 | Asymmetric modulation of electrical transport by piezotronic effect. a-c, Circuit diagram and corresponding strain-modulated band diagram of device with strain-free (**a-1** and **a-2**), tensile strain (**b-1** and **b-2**) and compressive strain (**c-1** and **c-2**). **d,** Schematic illustrations of the *I-V* curves of the device under different strain states.

References

1. Sze, S. M. & K, K. N. Physics of semiconductor devices, 3rd Edition. (Wiley, 2006).

2. Nahar, R., Singh, V., Sharma, A. Study of electrical and microstructure properties of high dielectric hafnium oxide thin film for MOS devices. *Journal of Materials Science: Materials in Electronics* **18**, 615-619 (2007).
3. Gilmer, D. *et al.* Compatibility of polycrystalline silicon gate deposition with HfO_2 and Al_2O_3/HfO_2 gate dielectrics. *Applied physics letters* **81**, 1288-1290 (2002).
4. Kang, S. *et al.* Effect of deposition conditions of poly $Si_{1-x}Ge_x$ films and Ge atoms on the electrical properties of poly $Si_{1-x}Ge_x$ (x= 0, 0.6)/HfO_2 gate stack. *Journal of Applied Physics* **94**, 4608-4613 (2003).
5. Hinkle, C., Fulton, C. *et al.* Enhanced tunneling in stacked gate dielectrics with ultra-thin HfO_2 (ZrO_2) layers sandwiched between thicker SiO_2 layers. *Applied Surface Science* **234**, 240-245 (2004).
6. Gerritsen, E. *et al.* Evolution of materials technology for stacked-capacitors in 65 nm embedded-DRAM. *Solid-State Electronics* **49**, 1767-1775 (2005).
7. Pan, C., Zhai, J., Wang, Z. L. Piezotronics and piezo-phototronics of third generation semiconductor nanowires. *Chemical Reviews* **119**, 9303-9359 (2019).
8. Wang, L., Wang, Z. L. Advances in piezotronic transistors and piezotronics. *Nano Today* **37**, 101108 (2021).
9. Zhu, L., Wang, Z. L. A perspective on piezotronics and piezo-phototronics based on the third and fourth generation semiconductors. *Applied Physics Letters* **122**, 250501 (2023).
10. Moučka, R. *et al.* Mechanical properties of bulk Sylgard 184 and its extension with silicone oil. *Scientific Reports* **11**, 19090 (2021).
11. Peng, J., *et al.* Stiff and tough PDMS-MMT layered nanocomposites visualized by AIE luminogens. *Nature Communications* **12**, 4539 (2021).
12. Meng, L. *et al.* Enhancing the performance of room temperature ZnO microwire gas sensor through a combined technology of surface etching and UV illumination. *Materials Letters* **212**, 296-298 (2018).
13. Tamm, I. Über eine mögliche art der elektronenbindung an kristalloberflächen. *Zeitschrift für Physik* **76**, 849-850 (1932).
14. Shockley, W., Pearson, G. Modulation of conductance of thin films of

semiconductors by surface charges. *Physical Review* **74**, 232 (1948).

15. Shockley, W. On the surface states associated with a periodic potential. *Physical Review* **56**, 317 (1939).
16. Nicollian, E., Brews, J. MOS Physics and Technology, John Wiley & Sons (1982).
17. Fu, J. *et al*. Optical measurement of the converse piezoelectric d_{33} coefficients of bulk and microtubular zinc oxide crystals. *Applied Physics Letters* **90**, 191 (2007).
18. Fan, H. *et al*. Template-assisted large-scale ordered arrays of ZnO pillars for optical and piezoelectric applications. *Small* **2**, 561-568 (2006).
19. Lee, Y. *et al*. Control of ZnO nanorod array alignment synthesized *via* seeded solution growth. *Journal of Crystal Growth* **304,** 80-85 (2007).
20. Scrymgeour, D., Hsu, J. Correlated piezoelectric and electrical properties in individual ZnO nanorods. *Nano Letters* **8**, 2204-2209 (2008).
21. Minaryjolandan, M. *et al*. Individual GaN nanowires exhibit strong piezoelectricity in 3D. *Nano Letters* **12**, 970 (2016).
22. Ke, T. *et al*. Sodium niobate nanowire and its piezoelectricity. *The Journal of Physical Chemistry C* **112**, 8827-8831 (2008).
23. Wang, J. *et al*. Piezoresponse force microscopy on doubly clamped $KNbO_3$ nanowires. *Applied Physics Letters* **93**, 223101 (2008).
24. Yu, Q. *et al.* Highly sensitive strain sensors based on piezotronic tunneling junction. *Nature communications* **13**, 778 (2022).
25. Liu, S. *et al.* Statistical piezotronic effect in nanocrystal bulk by anisotropic geometry control. *Advanced Functional Materials* **31**, 2010339 (2021).
26. Meng, J., Li, Z. Schottky-contacted nanowire sensors. *Advanced Materials* **32**, 2000130 (2020).
27. Won, D. *et al.* Transparent electronics for wearable electronics application. *Chemical Reviews* **123**, 9982-10078 (2023).
28. Xu, X. *et al.* Status and prospects of MXene-based nanoelectronic devices. *Matter* **6**, 800-837 (2023).
29. Han, X., Ji, Y., Yang, Y. Ferroelectric photovoltaic materials and devices. *Advanced Functional Materials* **32**, 2109625 (2022).

30. Zhao, L. *et al.* Combining triboelectric nanogenerator with piezoelectric effect for optimizing Schottky barrier height modulation. *Science Bulletin* **66**, 1409-1418 (2021).
31. Li, H. *et al.* Triboelectric-polarization-enhanced high sensitive ZnO UV sensor. *Nano Today* **33**, 100873 (2020).
32. Meng, J. *et al.* Triboelectric nanogenerator enhanced Schottky nanowire sensor for highly sensitive ethanol detection. *Nano Letters* **20**, 4968-4974 (2020).
33. Kim, W. G. *et al.* Triboelectric nanogenerator: Structure, mechanism, and applications. *ACS Nano* **15**, 258-287 (2021).
34. Zhang, S. L. *et al.* Electromagnetic pulse powered by a triboelectric nanogenerator with applications in accurate self‐powered sensing and security. *Advanced Materials Technologies* **5**, 2000368 (2020).
35. Lone, S. A. *et al.* Recent advancements for improving the performance of triboelectric nanogenerator devices. *Nano Energy* **99**, 107318 (2022).
36. Müller, K. *et al*. Microscopic and spectroscopic characterization of interfaces and dielectric layers for OFET devices. *Physica Status Solidi (a)* **205**, 600-611(2008).
37. Moore, J. *et al*. Combined conducting atomic force/scanning Kelvin probe microscope for investigating charge trapping on semiconductor surfaces. *APS March Meeting Abstracts* (2009).
38. Yang, R., Qin, Y., Dai, L., Wang, Z. L. Power generation with laterally packaged piezoelectric fine wires. *Nature Nanotechnology* **4**, 34-39 (2009).
39. Liu, Y. *et al*. Effect of hydrostatic pressure on the barrier height of Ni Schottky contacts on *n*-AlGaN. *Applied Physics Letters* **88**, 022109 (2006).
40. Liu, Y. *et al*. Effects of hydrostatic and uniaxial stress on the Schottky barrier heights of Ga-polarity and N-polarity *n*-GaN. *Applied Physics Letters* **84**, 2112-2114 (2004).
41. Zhang, Y., Liu, Y., Wang, Z. L. Fundamental theory of piezotronics. *Advanced Materials* **23**, 3004-3013 (2011).
42. Liu, K., Sakurai, M., Aono, M. Enhancing the humidity sensitivity of Ga_2O_3/SnO_2 core/shell microribbon by applying mechanical strain and its application as a

flexible strain sensor. *Small* **8**, 3599-3604 (2012).

43. Wendel, P. *et al.* Polarization dependence of ZnO Schottky barriers revealed by photoelectron spectroscopy. *Physical Review Materials* **4**, 084604 (2020).
44. Wu, W., Wang, Z. L. Piezotronics and piezo-phototronics for adaptive electronics and optoelectronics. *Nature Reviews Materials* **7**, 1-17 (2016).
